# Host-dependent impairment of parasite development and reproduction in the acanthocephalan model

**DOI:** 10.1186/s13578-022-00818-2

**Published:** 2022-05-31

**Authors:** Hanno Schmidt, Katharina Mauer, Thomas Hankeln, Holger Herlyn

**Affiliations:** 1 Anthropology, Institute of Organismic and Molecular Evolution (iomE), Johannes Gutenberg University Mainz, Mainz, Germany; 2 Molecular Genetics and Genomic Analysis, Institute of Organismic and Molecular Evolution (iomE), Johannes Gutenberg University Mainz, Mainz, Germany

**Keywords:** Gene expression, Reproduction, Energy metabolism, Immune response, Eel, Barbel, RNA-Seq, *Schistosoma*, Nematoda, Cestoda

## Abstract

**Background:**

A central question in parasitology is why parasites mature and reproduce in some host species but not in others. Yet, a better understanding of the inability of parasites to complete their life cycles in less suitable hosts may hold clues for their control. To shed light on the molecular basis of parasite (non-)maturation, we analyzed transcriptomes of thorny-headed worms (Acanthocephala: *Pomphorhynchus laevis*), and compared developmentally arrested worms excised from European eel (*Anguilla anguilla*) to developmentally unrestricted worms from barbel (*Barbus barbus*).

**Results:**

Based on 20 RNA-Seq datasets, we demonstrate that transcriptomic profiles are more similar between *P. laevis* males and females from eel than between their counterparts from barbel. Impairment of sexual phenotype development was reflected in gene ontology enrichment analyses of genes having differential transcript abundances. Genes having reproduction- and energy-related annotations were found to be affected by parasitizing either eel or barbel. According to this, the molecular machinery of male and female acanthocephalans from the eel is less tailored to reproduction and more to coping with the less suitable environment provided by this host. The pattern was reversed in their counterparts from the definitive host, barbel.

**Conclusions:**

Comparative analysis of transcriptomes of developmentally arrested and reproducing parasites elucidates the challenges parasites encounter in hosts which are unsuitable for maturation and reproduction. By studying a gonochoric species, we were also able to highlight sex-specific traits. In fact, transcriptomic evidence for energy shortage in female acanthocephalans associates with their larger body size. Thus, energy metabolism and glycolysis should be promising targets for the treatment of acanthocephaliasis. Although inherently enabling a higher resolution in heterosexuals, the comparison of parasites from definitive hosts and less suitable hosts, in which the parasites merely survive, should be applicable to hermaphroditic helminths. This may open new perspectives in the control of other helminth pathogens of humans and livestock.

**Supplementary information:**

The online version contains supplementary material available at 10.1186/s13578-022-00818-2.

## Background

Parasites might seem rare, but actually are very common. It is estimated that around 50% of all animal species live parasitically or at least go through parasitic life phases [1, 2]. Also, almost every animal species is being exploited by parasites [3], with humans and livestock being no exception. However, of the many possible hosts, parasite species use only a few to single ones [4], while they do not establish infections in other species. In case of complex life cycles, one may distinguish between higher-level hosts in which parasites mature and reproduce (definite or definitive hosts) and such hosts in which they might survive but usually are developmentally delayed or arrested (paratenic and accidental hosts) [5]. However, compared to parasitic infections of definitive hosts (e.g. [6, 7]), comparatively little is known about the molecular background of the mere survival of parasites in paratenic and accidental hosts. However, a better understanding of the molecular basis of host-dependent parasite plasticity promises clues for parasite control. This consideration prompted us to study the molecular underpinnings of host-dependent parasite plasticity in thorny-headed worms (Acanthocephala).

Acanthocephalans are gonochoric parasites with pronounced sexual dimorphism of body size [8]. They occur worldwide in the intestinal tracts of cartilaginous fishes (Chondrichthyes), ray-finned fishes (Actinopterygii), amphibians (Amphibia), sauropsids (Sauropsida), and mammals (Mammalia) [8]. As it seems, virtually every species of jaw-bearing vertebrates (Gnathostomata) can get infected, provided that intermediate hosts from jaw-bearing arthropods (Mandibulata) or parasitized gnathostomes belong to the diet. Humans infect themselves by taking up raw or insufficiently cooked hosts, which obviously played a greater role in prehistoric times than it does today (reviewed in [9]). In addition, acanthocephalans can cause major difficulties in human livestock including domestic pig [10], duck [11], and chicken [12]. Yet, the most comprehensive data on detrimental consequences of acanthocephaliasis is available for cultivated fish. Thus, acanthocephalans quite regularly contribute to the parasite fauna in marine aquacultures [13–15]. Infections are further reported for limnocultures of brown trout (*Salmo trutta fario*), tambaqui (*Colossoma macropomum*), pirarucu (*Arapaima gigas*) and Nile tilapia (*Oreochromis niloticus*), amongst others [16–19], where high intensities can cause reduced growth, weakening, and emaciation [20, 21]. Deformation and death of fishes are additional regular consequences of infections with acanthocephalans [13, 16]. Acanthocephaliasis is even considered the main obstacle to successful aquaculture in the mainland of countries such as Brazil. Here, extreme intensities of up to several hundred worms per fish and prevalences of up to 100% can cause severe to total economic losses [20, 22–26].

Life-threatening courses of acanthocephaliasis result from obstruction and peritonitis [27–29]. But acanthocephalans also harm their hosts at a lower-threshold level, namely by injuring various tissues, foremost the intestinal wall. In particular, the action of their mostly hooked anterior holdfast [30, 31] induces bleeding, inflammatory reactions, and necrosis [32–35]. In addition, the worms damage their hosts by depriving or withdrawing nutrients and minerals which the gutless worms take up via surface [36–40].

Carbohydrates enter the acanthocephalan body via the same route and are simultaneously metabolized in aerobic and anaerobic pathways [41–43] or stored into glycogen particles [31, 44, 45]. It is plausible to assume that energy demand is generally high in acanthocephalans considering the need to produce many offspring for keeping the life cycle running. Indeed, the larger female produces thousands up to millions of eggs in multiple smaller ovaries floating in the trunk body cavity [46–49]. Although smaller in size [8, 50], male morphology is also tailored to reproduction: a large part of the trunk body cavity is filled by the two tandem-arranged testes, which are larger in cases of elevated levels of sperm competition [51]. Additionally contained are one bigger or several smaller glands for proteinaceous secretion [52]. After copulation, males apply this so-called cement to the female rear end, thus sealing the female genital tract and preventing subsequent mating [53]. Evidence of increased intra-male competition also comes from the occasional capping of the male posterior end, so that the affected male is at least temporarily excluded from reproduction [54]. Not least, males seem to play a more active role in mating than females [55–57].

The perhaps best-studied acanthocephalan is *Pomphorhynchus laevis* (Zoega in Müller, 1773) Monticelli, 1905 (Palaeacanthocephala). In fact, the first genome and transcriptome assemblies for acanthocephalans are available for this taxon [58]. Additionally, extensive data on the morphology, ecology and life history exist for *P. laevis* (e.g. [31, 59, 60]). It is a common parasite of ray-finned fishes in Western Palearctic freshwaters. Especially, several salmonid and cyprinid fishes can get infected when taking up gammarids serving as intermediate hosts [24]. One of its definitive hosts is the common barbel (*Barbus barbus*) [61, 62] whereas *P. laevis* usually does not sexually mature and reproduce in the European eel (Anguillidae: *Anguilla anguilla*) [63]. Thus, the worms in the eel remain smaller than in the barbel, and the degree of (reverse) sexual dimorphism in body size is not as pronounced in the worms from eel as in those from barbel: here the females can be up to eight times as voluminous as the males [8, 64].

However, the deeper causes of developmental plasticity of *P. laevis* in various vertebrate hosts are largely unknown, as is the case with parasites in general. To shed light on host-dependent parasite maturation in the acanthocephalan model, we quantitatively analyzed the transcriptomes of male and female *P. laevis* specimens from common barbel and European eel. We discuss the findings in the light of basic evolutionary processes and implications for the development of new drugs for parasite control.

## Results

### Survey on samples and transcriptomes

The *P. laevis* specimens from the eel were overall smaller and their inverse sexual dimorphism appeared to be less pronounced than in their conspecifics from barbel. In addition, worms from barbel were fully turgescent and mature, whilst they were saggy and developmentally delayed when originating from eel. For example, male worms from eel had poorly developed testes while these were normally developed in males from barbel. In addition, cement glands were well developed in males from barbel. Furthermore, the hind end of female worms from barbel partially carried copulatory caps. To elucidate the molecular background of this morphological plasticity, RNA-Seq was carried out on five worms per sex from each of the two fish species eel and barbel (total N = 20). Sequencing of the *P. laevis* transcriptomes resulted in about 651.3 million reads with an average of 32.6 million reads per sample. Out of these, 99.2% passed adapter clipping and quality processing. On average 95.9% of the reads mapped to the reference transcriptome [58] (Additional file 1: Table S1). Transcript quantification with RSEM revealed that 18,740 genes had relevant read numbers (≥ 10) in at least one sample. This corresponds to 65.1% of all genes present in the reference transcriptome representing male, female and juvenile worms [58].

### Distinct transcriptome profiles in male and female acanthocephalans from different hosts

**Fig. 1:**
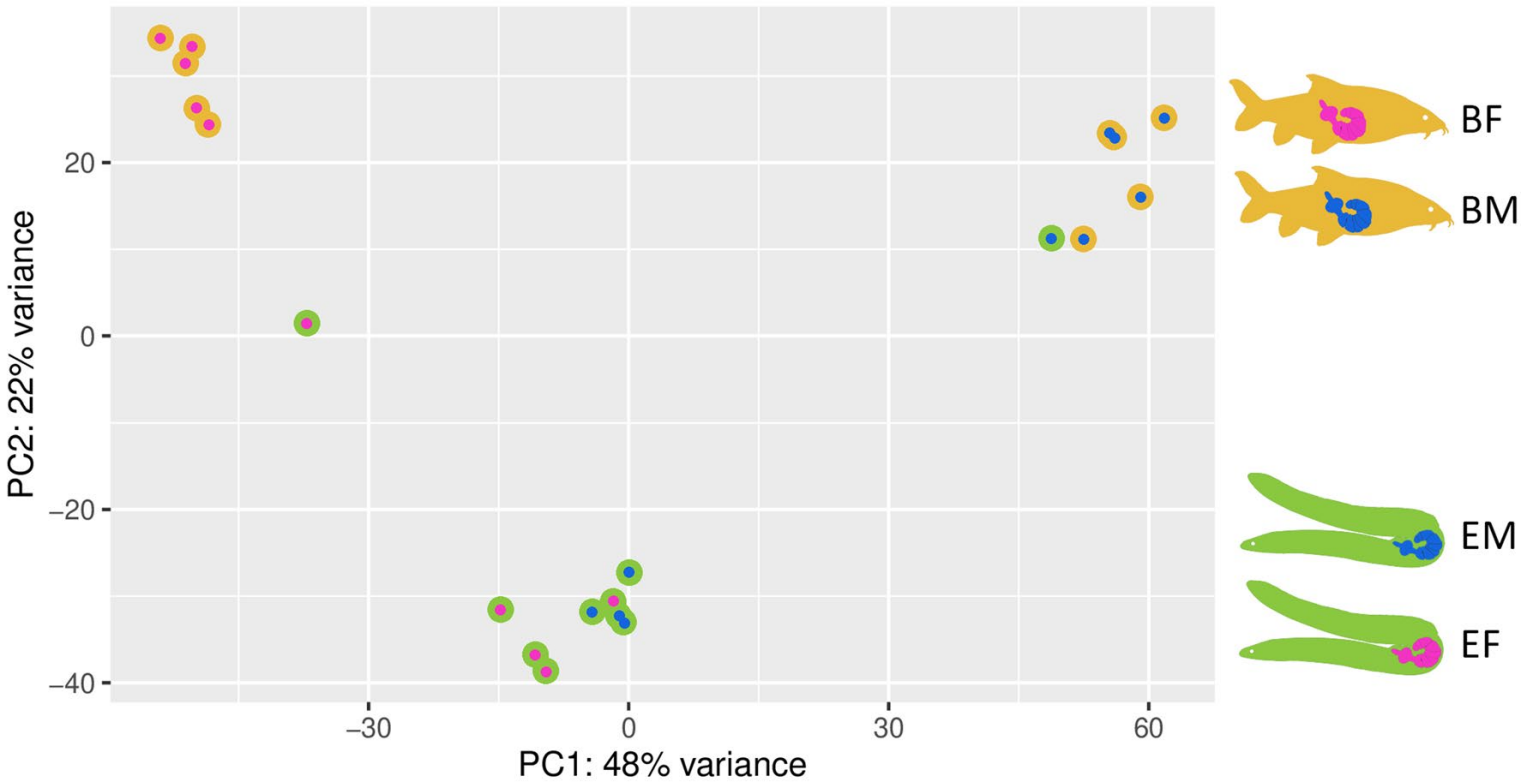
Principal Component Analysis of transcriptome-wide abundance patterns in dependence of sex and host. Principal Component 1 (PC1), explaining the majority of variance, separates the parasite sexes in barbel. Principal Component 2 (PC2) separates worms from different hosts. Dots are colored based on groups (female/male worms from barbel/eel). Each dot represents one worm, with five worms per group. Graphical symbols at the right refer to the host the worms were taken from (barbel, eel) with the sex of the worms indicated by the coloring blue for male and pink for female worms (BF: female worms from barbel, BM: male worms from barbel, EM: male worms from eel, EF: female worms from eel)

**Fig. 2:**
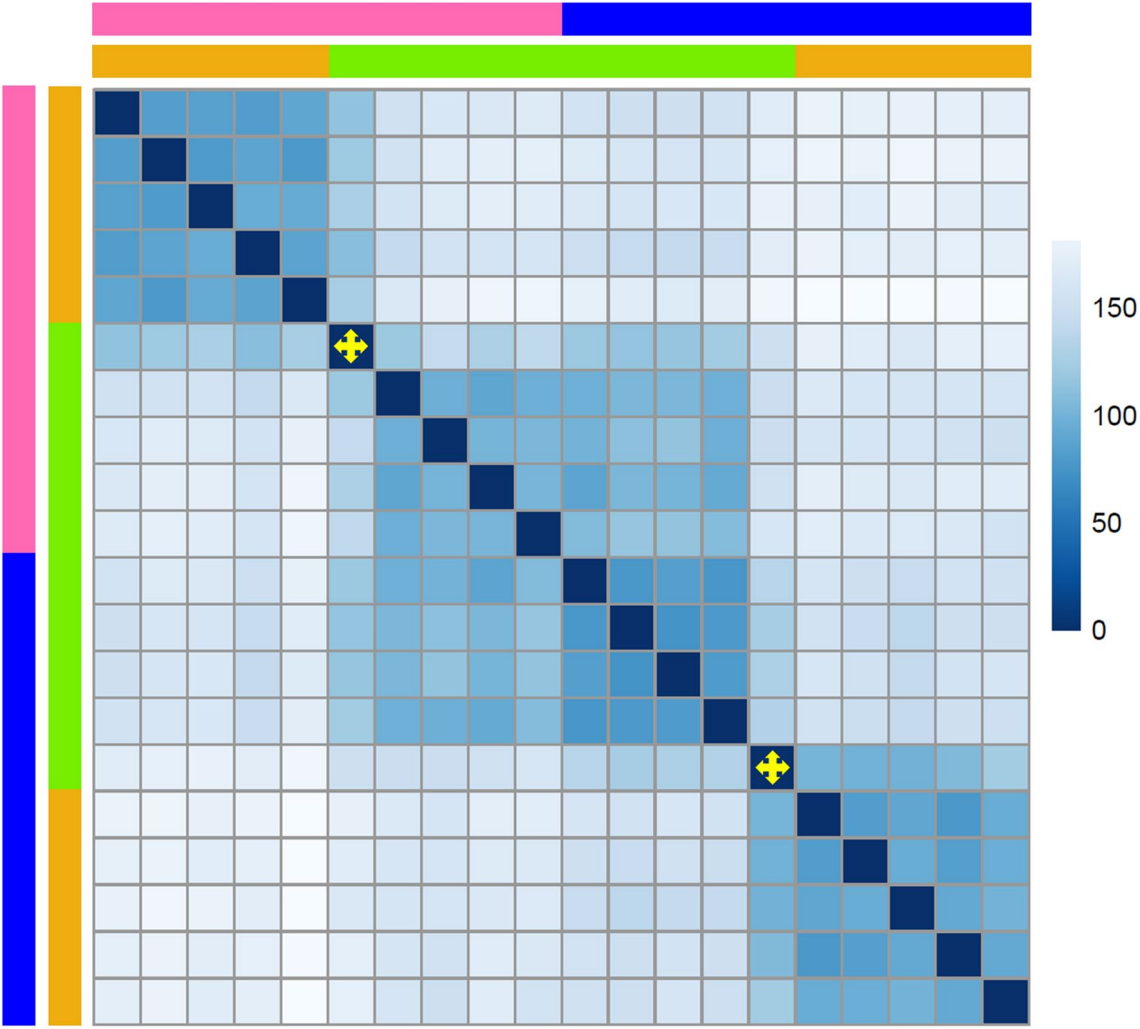
Sample-to-sample distances. Heat map of distances between each of the 20 samples to every other one. The bars on top and left of the plot indicate sample origin: blue is for male worms, pink is for female worms, orange is for worms from barbel and green is for worms from eel. The color key on the right assigns sample distances ranging from dark blue for no difference to white for completely different. The yellow four-way arrows highlight the two outlier samples as seen in the PCA which have an intermediate state between worms of the two hosts

Transcriptome profiles of all 20 samples were used for a principal components analysis (PCA) to assess the overall relationships between the four worm sex to host species combinations. Principal component 1 (PC1) explained 48% of the variance and primarily segregated datasets representing male and female *P. laevis* specimens excised from common barbel (Fig. 1). Compared to this, the samples of male and female worms from European eel were less clearly separated along PC1. Actually, male and female worms from the eel clustered together in between the sex-specific clusters from barbel along PC1. With 22%, the explanatory power of principal component 2 (PC2) was much smaller, thereby basically setting apart worms from eel and barbel. In the scatterplot, the pattern described was reflected in one cluster representing female worms from barbel, a second one containing male specimens from barbel, and a third cluster comprising almost all eel-born worms regardless of their sex. The exception from the latter were datasets generated from a single male and one female, which approximate the clusters representing worms with corresponding sex from barbel. These two samples also were intermediary in an overall sample-to-sample distance matrix (Fig. 2), indicating advanced development compared to their conspecifics from eel, but not reaching the degree of maturity present in males and females from barbel. Besides, the distance matrix confirmed the overarching pattern of three main clusters, one containing female worms from barbel, one including male worms from barbel, and one containing the samples from eel (Fig. 2).

**Fig. 3:**
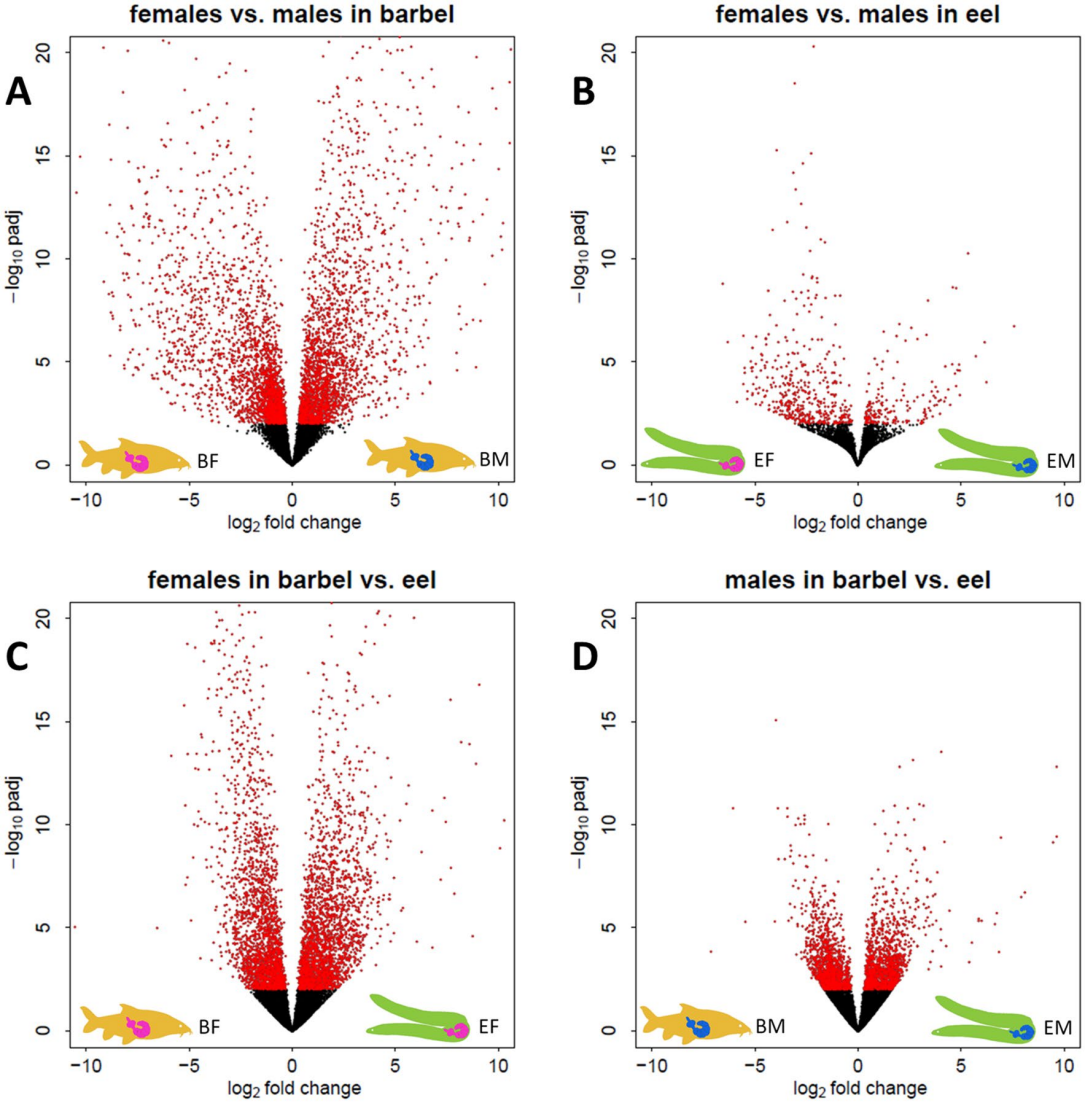
Transcriptome-wide differences in transcript abundances. Shown are log_2_-fold change against -log_10_ of the adjusted p-value (padj) for all genes. **A** Volcano plot of male vs. female worms parasitizing barbel. **B** Volcano plot of male vs. female worms parasitizing eel. **C** Volcano plot of female worms parasitizing barbel and eel. **D** Volcano plot of male worms parasitizing barbel and eel. Each dot represents a gene. Red dots indicate genes showing significant expression differences (padj ≤ 0.05), black dots indicate genes showing non-significant differences between the groups compared. Differences are more pronounced between male and female acanthocephalans from barbel and between female worms from barbel and eel than in the other pairs of comparison

**Fig. 4:**
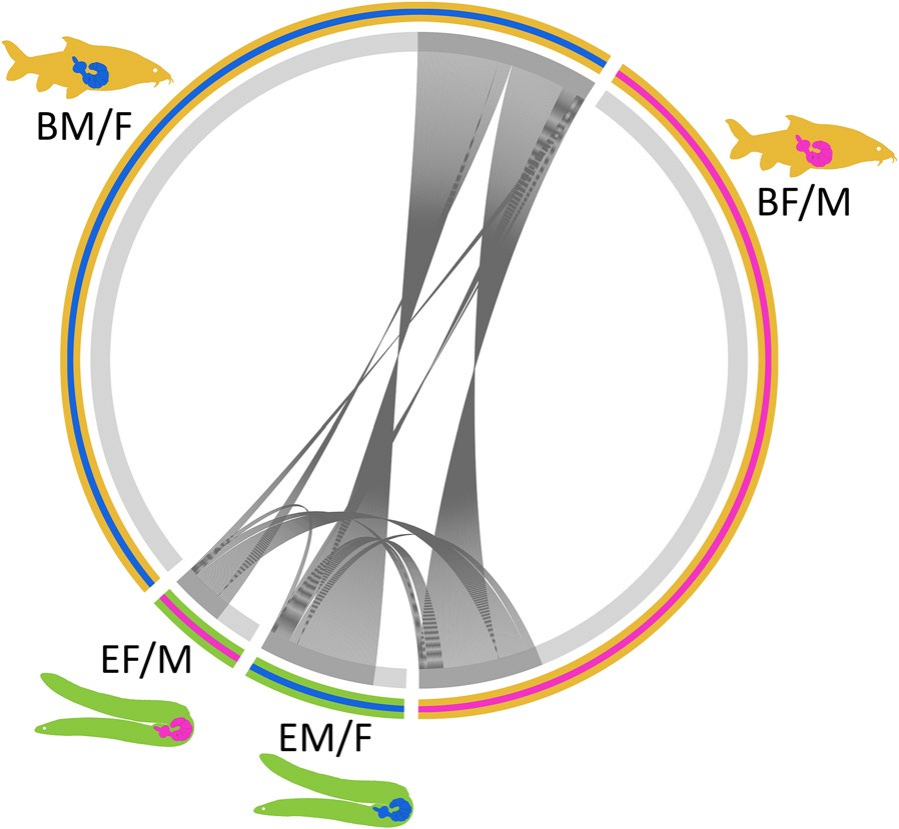
Similarities and differences between sets of genes differing in transcript abundances. Coloration refers to genes with significantly increased transcript abundances in male vs. female acanthocephalans from barbel (BM/F), females vs. males from barbel (BF/M), females vs. males from eel (EF/M), and males vs. females from eel (EM/F). Circular sections are scaled to the number of genes with elevated transcript numbers in a particular pair of comparison. Grey strings connect corresponding genes across different pairs of comparison. Many more genes exhibit differential transcript abundances between male and female worms from barbel than between their counterparts from eel. Connections between male and female acanthocephalans from the same host species relate to different genes with annotation overlaps in the reference (*D. melanogaster*)

**Fig. 5:**
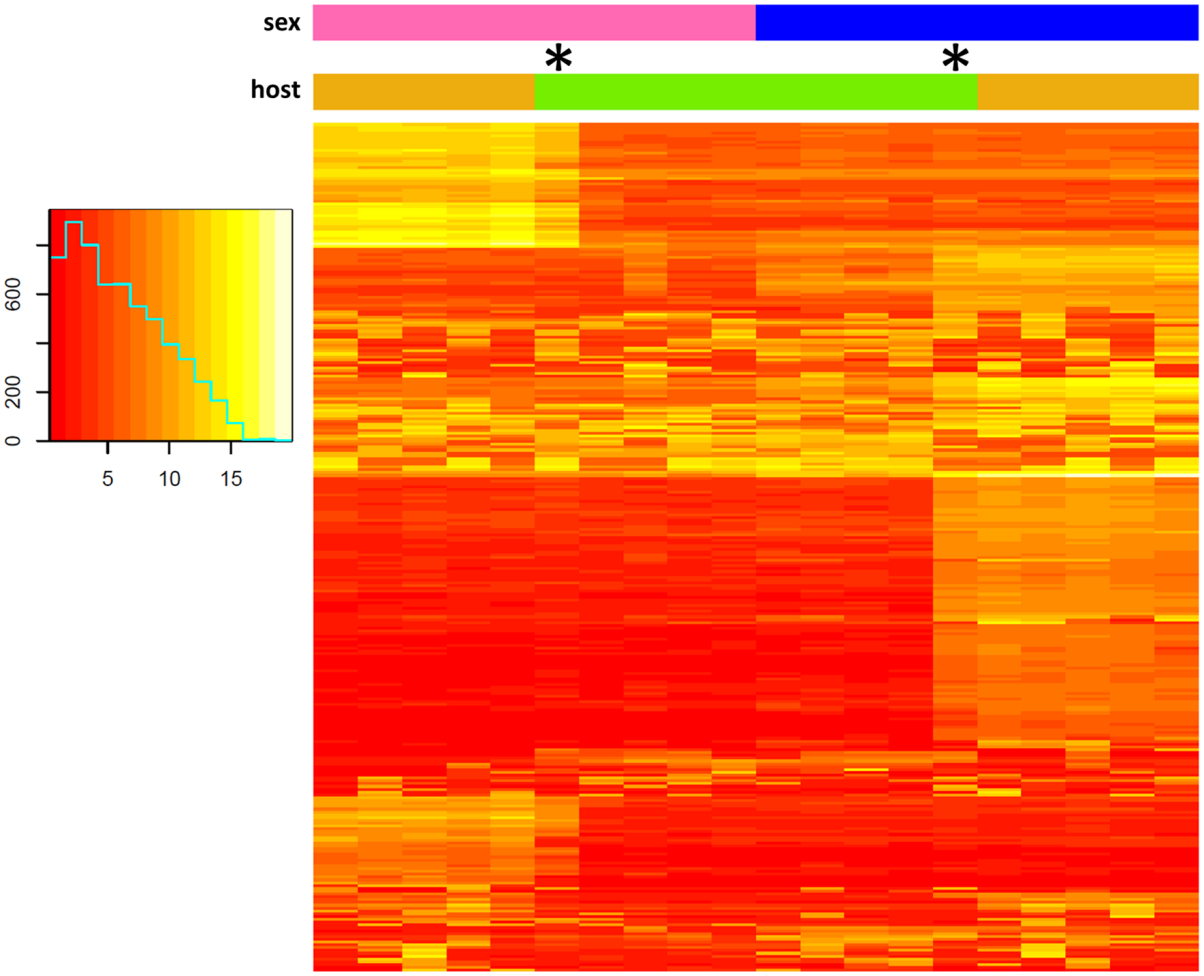
Gene clustering. The heatmap refers to the 300 genes with the highest variance of transcript abundance across samples. Clustering was used for rows, not for columns. Colored bars on top indicate sample origin: blue is for male worms, pink is for female worms, orange is for worms from barbel and green is for worms from eel. Asterisks indicate the two outlier samples showing an overall intermediate state between worms from both hosts, with individual genes alternately resembling expression patterns of one group or the other

Comparative analysis of transcript abundances between male and female worms from single hosts (DESeq2) supported a tentative sexual differentiation breakdown in acanthocephalans excised from eel. While transcript numbers of 6131 genes significantly differed between male and female worms from barbel, only 1326 genes had sex-dependent transcript abundances in worms from eel (≈ 1/5). In addition, we observed higher overall log fold-changes between parasites from barbel relative to comparisons between worms from eel, as illustrated in the respective volcano plots (Fig. 3A, B). Furthermore, variation in transcript abundances were more pronounced between *P. laevis* females from barbel vs. eel than between male worms from both hosts (Fig. 3C, D). Matching of genes showing significantly higher transcript abundance in pairs of comparison underscored more pronounced sexual differentiation of *P. laevis* in barbel than eel. The corresponding circus plot further demonstrates that the genes with differential transcript abundances between males and females from the eel were largely a subset of the genes showing differential transcript abundances between males and females from the barbel (Fig. 4; Additional file 2: Table S2). Lastly, clustering analysis of the 300 genes with the highest variance of transcript abundances across samples revealed mixed rather than clear-cut profiles for male and female worms from the eel, relative to their conspecifics from barbel. The heatmap additionally underlined the intermediary state of the two outlier samples mentioned above (Fig. 5).

### Functional involvements of genes with differentially abundant transcripts between male and female acanthocephalans from barbel

**Fig. 6:**
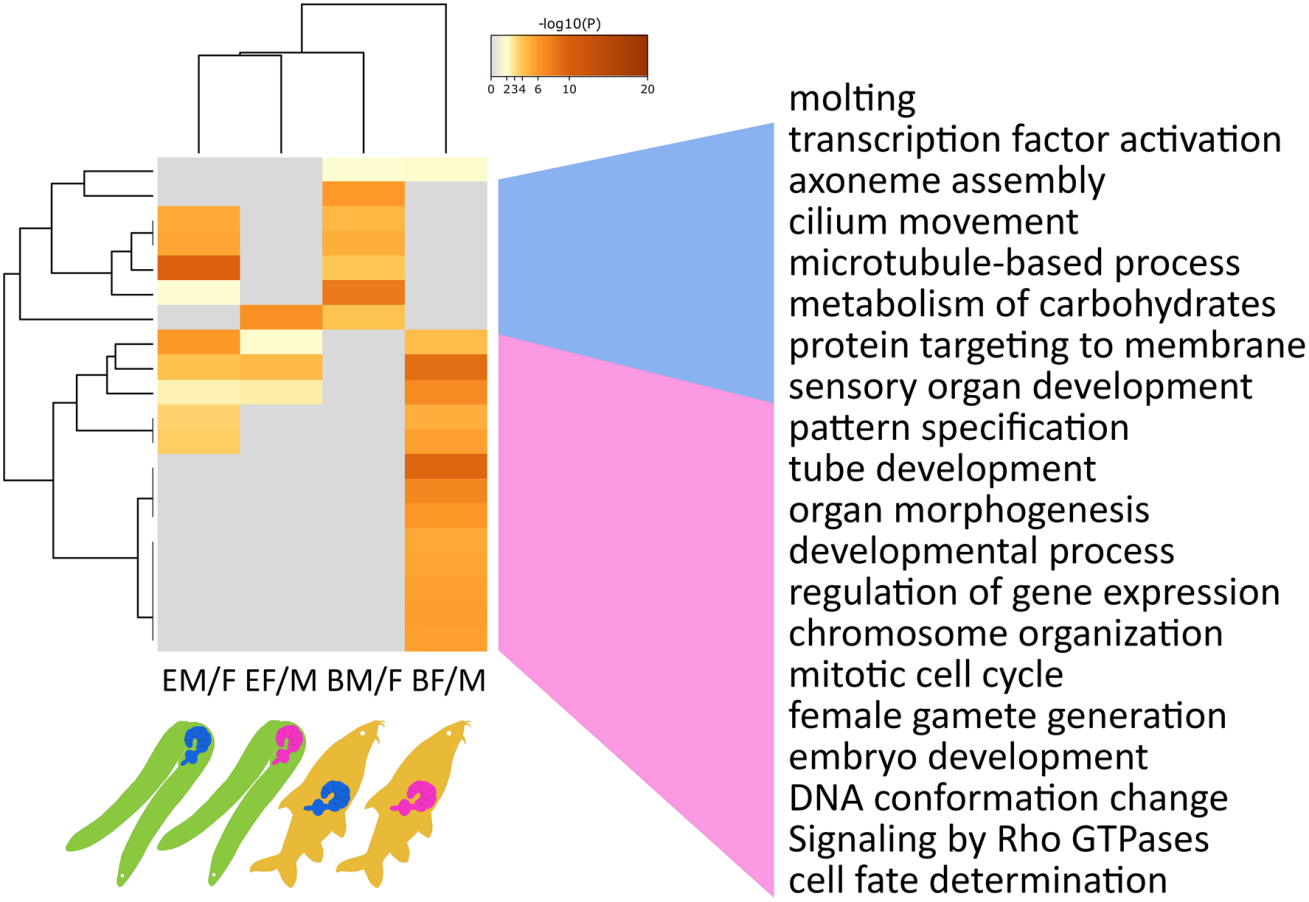
Principal patterns in functional annotations of genes showing different transcript abundances. The heatmap refers to the 20 functional annotation terms with highest significance for enrichment between groups in the comparison of male vs. female worms from single hosts. Blue and pink trapezoids connect to functional annotation term clusters separating male (BM/F) and female (BF/M) worms from barbel. No such clear separation emerges from the heatmap for males from eel (EM/F) and females from eel (EF/M)

Functional enrichment analysis with Metascape corroborated that sexual differentiation was more advanced in worms from barbel than eel. Out of the 20 functional categories with highest significance, 19 categories were either enriched in genes showing increased transcript abundances in the male vs. female or female vs. male comparisons of worms from barbel (Fig. 6). Of these, 13 categories were enriched in genes with increased transcript levels in female worms, with eight categories being relatable to development (“tube development”, “organ morphogenesis”, “sensory organ development”, “developmental process”, “pattern specification”, “cell fate determination”) and reproduction (“female gamete generation”, “embryo development”). The other five of the above 13 categories were less clearly assignable to an overarching theme but under the premise that development and reproduction are predominant functions, they seem quite fitting (“mitotic cell cycle”, “regulation of gene expression”, “chromosome organization”, “DNA conformation change”). Six additional categories were significantly enriched in genes exhibiting higher transcript abundances in male vs. female worms from barbel, three of which being attributable to sperm production (“axoneme assembly”, “cilium movement”, “microtubule-based process”). One additional category referred to “metabolism of carbohydrates”. In contrast, only seven categories were sex-specifically enriched in genes with divergent transcript levels between worms from eel, with four of them corroborating patterns described for worms from barbel.

**Fig. 7:**
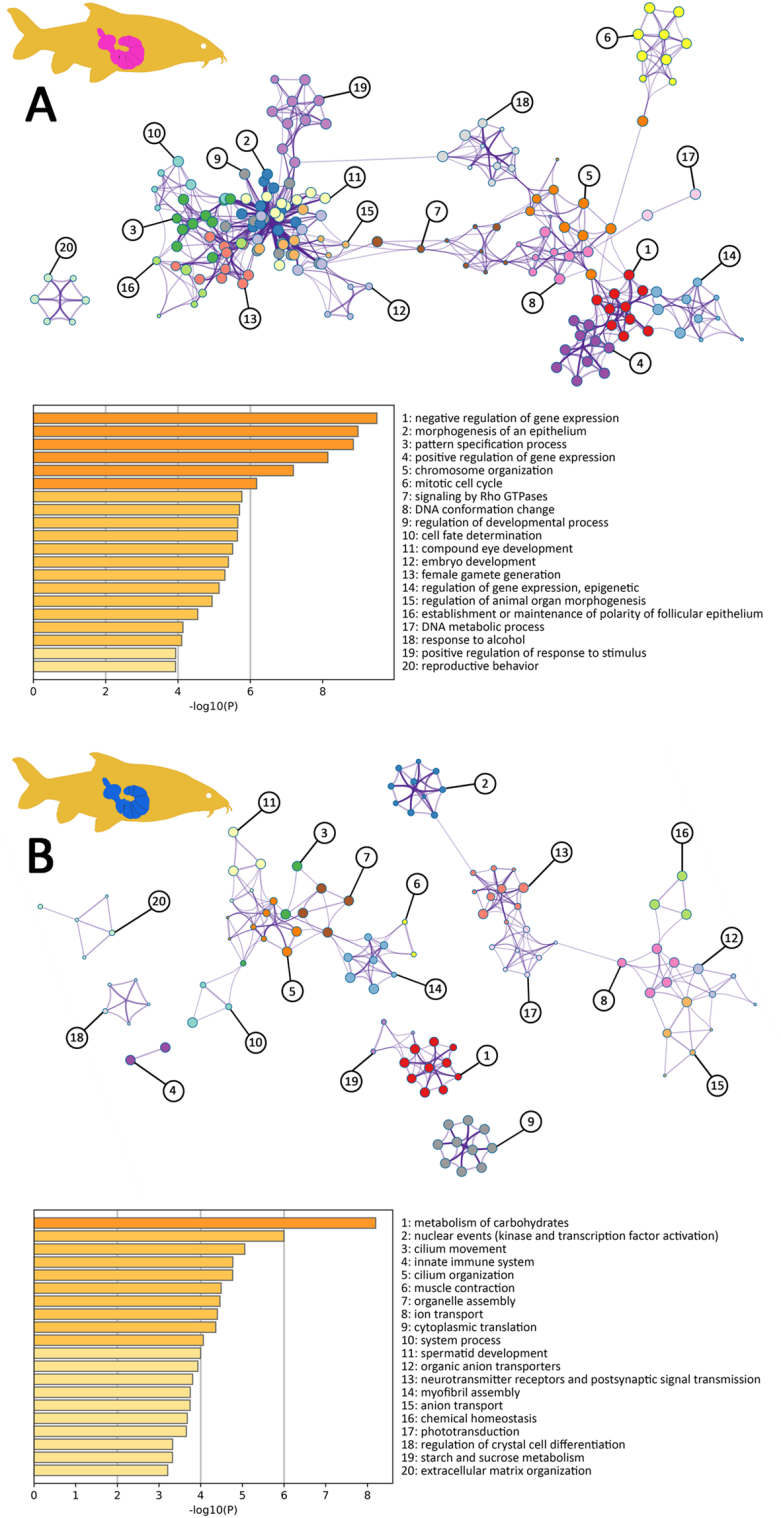
Functional differences for genes having differential transcript levels in *P. laevis* males and females from barbel. **A** Functional terms enriched in genes exhibiting higher transcript numbers in female vs. male worms from barbel (BF/M). **B** Functional terms enriched in genes exhibiting higher transcript numbers in male vs. female worms from barbel (BM/F). Nodes of the networks in **A** and **B** represent GO term clusters, grouped and colored by overarching descriptions. Clusters are specified according to their numbering along with statistical confidence levels (log10 p-values) in the lower section of each graphic

Gene Ontology (GO) terms enriched in genes having differential transcript abundances were plotted as networks and functionally clustered with Metascape. Out of the 20 highest scoring GO clusters in genes with significantly more transcripts in female vs. male acanthocephalans from barbel, at least ten underlined involvements in development and reproduction (see nos. 2, 3, 9, 10, 11, 12, 13, 15, 16, and 20 in Fig. 7A). They were all contained in the largest connected component which additionally included eight GO clusters which might indirectly connect to development (nos. 1, 4, 5, 6, 7, 8, 14, 17). The network further highlighted an increased importance of response to stimulation in female parasites from barbel (nos. 7, 18, 19). With “response to alcohol” one cluster related to energy metabolism. Signaling was reflected by the GO cluster “signaling of Rho GTPases”.

The GO clusters representing genes which had elevated transcript levels in male vs. female worms from barbel spread across several subnetworks (Fig. 7B). Still, the composition of the largest connected component underscored sperm production as a major function in males from barbel (nos. 3, 5, 7, 11). This was most evident in the GO cluster “spermatid development”, but “cilium movement” and “cilium organization” would be in accordance with sperm production as well. Likewise, “organelle assembly” could be an indirect hint to male gametogenesis. Furthermore, GO clusters relating to muscular assembly and contraction (nos. 6, 14) suggest particular importance of activity for male acanthocephalans from barbel. Corresponding evidence was based on the genes encoding troponin T, myosin heavy chain, tropomyosin 2, myosin alkali light chain 1 and others, out of which only the gene tropomyosin 2 exhibited higher transcript abundance in males vs. females from eel. The majority of GO term clusters in a second subnetwork illustrated connections to stimulation and ion transport as exemplified by “neurotransmitter receptors and postsynaptic signal transmission” (nos. 8, 12, 13, 15, 17). Furthermore, the enhanced relevance of carbohydrate metabolism in male acanthocephalans was confirmed in the GO cluster network (nos. 1, 19). In fact, transcript levels of eleven genes of the glycolysis/gluconeogenesis pathway were raised in male vs. female worms from barbel (Additional file 1: Fig. S1). Additional genes with higher transcript numbers in males vs. females from barbel related to the wider context of energy metabolism and glycolytic side pathways (Table 1). Not least, the GO cluster “innate immune system” was enriched in genes with high-abundance transcripts in male *P. laevis* specimens (no. 4).

**Table 1:** Carbohydrate metabolism genes showing increased transcript levels in male vs. female worms from barbel

Gene	Log2 fold change	Adjusted p-value	Function
Aldolase 1*	8.12	5.5E−11	Developmental stage-specific or tissue -specific sugar-phosphate metabolisms
Hexokinase A*	6.63	3.6E−09	Glucose homeostasis
Glyceraldehyde 3 phosphate dehydrogenase 2 *	5.47	2.4E−10	Glucose homeostasis
Phosphoglycerate kinase*	5.25	8.6E−11	Gluconeogenesis
NUCB1	3.84	2.3E−17	Carbohydrate metabolic process
Pyruvate kinase*	2.00	1.7E−17	Muscle development, glycolysis and glucose homeostasis
Glycogenin	1.62	9.1E−07	Glycogenin glucosyltransferase activity
Glycerol-3-phosphate dehydrogenase 1	1.37	2.7E−05	Enzymatic oxidation of glycerol-3-phosphate to dihydroxyacetone phosphate
Glycogen phosphorylase	1.29	2.3E−06	Important allosteric enzyme in carbohydrate metabolism
Trehalose-6-phosphate synthase 1	1.26	2.3E−04	Enzymatic production of T6P using glucose-6-phosphate and UDP-glucose
N-acetylglucosamine kinase	1.20	3.6E-−04	Carbohydrate phosphorylation
Pyruvate carboxylase	1.18	2.7E−05	Gluconeogenesis
Hexosaminidase 1	1.18	8.3E−05	Protein deglycosylation and rhodopsin biosynthesis
Poly(ADP-ribose) glycohydrolase	1.16	9.1E−09	Degrades poly(ADP-ribose) to mono(ADP-ribose)
Glucosidase 2 α subunit	1.15	9.3E−05	Glucan 1,3-alpha-glucosidase activation; N-glycan processing
Oscillin	1.11	2.7E−05	Glucosamine-6-phosphate deaminase activation
Phosphoenolpyruvate carboxykinase 2*	1.06	1.3E−03	Manganese ion binding activation and phosphoenolpyruvate carboxykinase (GTP) activation
4-alpha-glucanotransferase	1.02	1.1E−09	4-alpha-glucanotransferase activation and amylo-alpha-1,6-glucosidase activation
Malate dehydrogenase 1	1.01	3.7E−05	Interconversion of malate and oxaloacetate
Glycogen synthase	1.01	1.3E−04	Enzymatic linkage of glucose monomers into glycogen

Genes up-regulated in male worms from barbel were found enriched with the GO term “metabolism of carbohydrates”. Of those, the 20 genes with the highest (and significant) log2 fold change of transcript abundances are shown here. The functions indicated refer to annotations of homologs in *Drosophila melanogaster* (flybase.org). Genes marked with an asterisk are part of the KEGG pathway “glycolysis/gluconeogenesis” (see Additional File 1: Fig. S1)

### Functional entanglements of genes having differential transcript levels between male and female acanthocephalans from eel

In eel, enrichment patterns of functional categories were far less distinct between male and female worms than in their conspecifics from barbel. In fact, merely 10 categories out of the 20 ones with lowest adjusted p-values were relating to genes with sex-specific transcript levels in worms from eel (Fig. 6). Only a single category was specifically enriched in genes with higher transcript abundances in female vs. male acanthocephalans from this host (“protein targeting to membrane”). Three additional ones occurred at increased frequencies in male and female transcriptomes. This was due to different genes in *P. laevis* having corresponding homologues and thus identical annotations in the reference species, *D. melanogaster*. Either way, the respective GO categories stressed the high relevance of developmental processes in male and female *P. laevis* specimens from the eel: “pattern specification”, “tube development”, and “organ morphogenesis”. Of the six categories with enrichment in genes having elevated transcript levels in male worms from eel, two were shared with female worms from barbel and four with male worms from barbel. We take this relationship as an indication of delayed development of male worms from eel toward the adequate phenotype. In line with this, only three categories enriched in genes with higher transcript abundances in male vs. female worms from eel might relate to reproduction: “axoneme assembly”, “cilium movement”, and “microtubule-based process”. Another category pointed to an increased relevance of “metabolism of carbohydrates”.

**Fig. 8:**
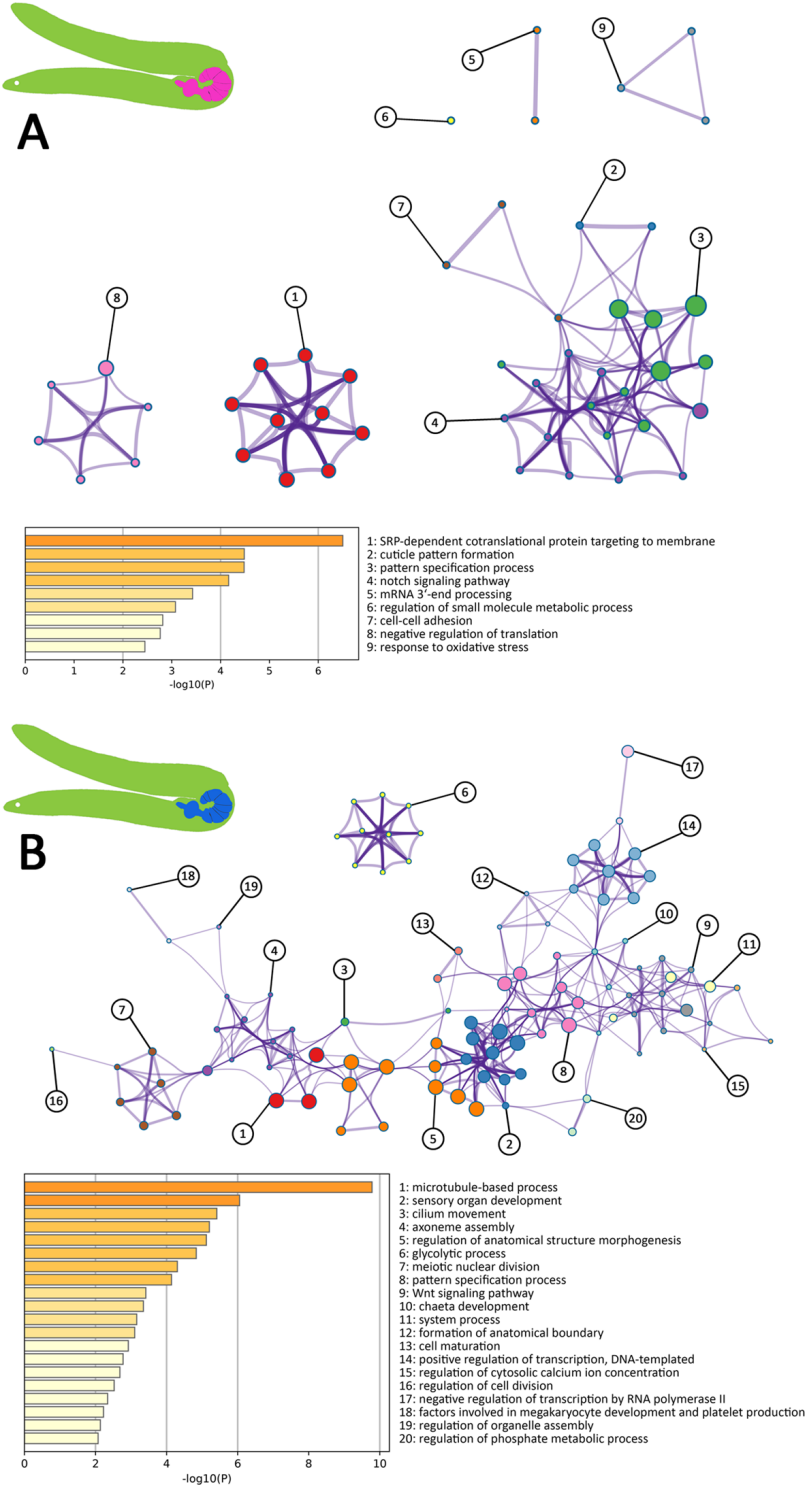
Functional differences for genes having differential transcript levels in *P. laevis* males and females from eel. **A** Functional terms enriched in genes exhibiting higher transcript numbers in female vs. male worms from eel (EF/M). **B** Functional terms enriched in genes exhibiting higher transcript numbers in male vs. female worms from eel (EM/F). Nodes of the networks in **A** and **B** represent GO term clusters, grouped and colored by overarching descriptions. Clusters are specified according to their numbering along with statistical confidence levels (log10 p-values) in the lower section of each graphic

Strikingly, in genes having more transcripts in *P. laevis* females vs. males from eel only nine GO clusters had enrichment p-values < 0.01, and these were less connected in the corresponding network than the clusters for their counterparts in female vs. male *P. laevis* specimens from barbel. One of the corresponding ‘female’ clusters in eel related to “response to oxidative stress” (no. 9). Four further GO clusters (nos. 1, 2, 3, 4) potentially referred to development once more (Fig. 8A), and an additional one hinted to the notch signaling pathway (no. 4). However, none of the respective GO clusters indicated a link to reproduction. In the genes having more transcripts in male vs. female worms from eel, relevance for reproduction emerged from enrichment of the GO cluster “meiotic nuclear division” (no. 7). Indirect hints for an involvement in sperm production and storage might be seen in GO clusters such as “cilium movement”, “cell maturation”, and “regulation of organelle assembly” (Fig. 8B: nos. 1, 3, 4, 13, 16, 19). Cluster analysis further underscored frequent involvements in developmental processes in males from eel (nos. 2, 5, 8, 9, 10, 12), as exemplified by “sensory organ development” and “regulation of anatomical structure morphogenesis“. Enrichment of developmentally relevant GO clusters in genes with increased transcript abundances in male vs. female acanthocephalans from eel was accompanied by high coherence of the network, when compared to the corresponding reconstruction for female worms from the eel. Furthermore, with “glycolytic process” (no. 6), energy metabolism was signified in high-abundance transcripts of males from the eel, as was the “Wnt signaling pathway” (no. 9).

### Complementary evidence for developmental halt in acanthocephalans parasitizing the eel

The majority of significantly enriched GO term clusters (84.1%, Figs. 7 and 8) were reproduced in BiNGO analyses (Additional file 1: Figs. S2–S5), thus underlining robustness of the findings above: developmental delay up to arrest in worms from eel vs. sexual maturation and reproduction in their conspecifics from barbel. The same pattern emerged when focusing on single genes. Specifically, out of 24 genes which had raised transcript levels in females vs. males from barbel and were involved in oocyte differentiation, only a single one showed a corresponding pattern in worms from eel. In addition, of the 21 spermatid development genes that exhibited higher transcript numbers between male vs. female worms from barbel merely seven were reproduced in the comparison of males and females from eel (Table 2). Comparing worms of the same sex but from different hosts underscored that acanthocephalans from the eel were less mature. Out of the 24 oocyte differentiation genes mentioned above, 19 had reduced transcript numbers in females from the less suitable vs. the definitive host, and none had more transcripts in the same pair of comparison. In males, transcript numbers were decreased in four out of the abovementioned 21 spermatid development genes in the eel vs. barbel comparison. Elevated transcript levels occurred in three genes in this comparison (Table 2). This might indicate particularly severe disruption of female physiology in the eel whilst male acanthocephalans seem to cope better with the less suitable environment provided by this host. The same could be reflected in genes implicated in cell cycle control and organ morphogenesis that displayed higher transcript levels in female acanthocephalans from eel vs. barbel (Additional file 1: Fig. S6). Developmental delay of female *P. laevis* specimens from eel vs. barbel was also apparent in the category “GTP hydrolysis and joining of the 60S ribosomal subunit” receiving highest significance for enrichment in this pair of comparison (Additional file 1: Fig. S7). Strikingly, transcripts of 58 or 79% of the 73 genes contained in the latter were found to be more numerous in female acanthocephalans from eel vs. barbel. These included several genes coding for subunits of five of the six eukaryotic initiation factors (eIF1-6). Increased translational activity was also suggested by BiNGO analysis of genes having increased transcript abundances in female vs. male worms from eel (Additional file 1: Fig. S4). Lastly, the genes showing lowered transcript numbers in males from eel vs. barbel converged to fewer functional categories (Additional file 1: Fig. S8) than those with increased abundance in the same pair of comparison (Additional file 1: Fig. S9). In the latter group of genes, functional categories “response to wounding” and “apoptosis” might point to stress response in male worms parasitizing eel.

**Table 2:** Genes implicated in gametogenesis

Involved in “oocyte differentiation” and higher transcript abundances in female vs. male worms from barbel	Involved in “spermatid development” and higher transcript abundances in male vs. female worms from barbel
14-3-3zeta	Act5C ↑
aPKC ↓	alphaTub84D*
baz ↓	Bug22* ↓
BicD ↓	Cds
Dcr-1	ctp ↑
dlg1 ↓	Diap1
Hsp83 ↓	didum*
l(2)gl	Dlc90F ↓
lic ↓	Fmr1
lost ↓	gudu ↓
Moe	jar
mus301 ↓	klhl10
orb* ↓	Klp59D* ↓
pAbp ↓	mfr*
pasha ↓	mia*
piwi ↓	Nap1
Pka-C1 ↓	Npc1a ↑
Rbfox1 ↓	Osbp
Rok ↓	Past1
sll ↓	sns
spn-E ↓	TTLL3B*
tsu ↓	
tud	
wbl ↓	

Shown are genes implicated in sex-specific gamete generation that showed differential transcript abundances in either male or female *P. laevis* specimens from barbel. Asterisks highlight genes with higher transcript abundances in corresponding pairs of comparison between worms from the eel. Arrows give the direction of differential transcript levels between male worms from barbel and eel and female worms from barbel and eel

## Discussion

Based on RNA-Seq data we have shown that sexual differentiation of the acanthocephalan *P. laevis* from a definitive host, common barbel, associates with pronounced transcriptomic signatures, while such signatures are much weaker in worms from eel. Especially, pathways relating to energy metabolism and reproduction appeared to be disturbed in worms from eel.

### Transcriptomic signatures of sexual maturation in acanthocephalans from a definitive host, barbel, have correlates in morphological and life history parameters

Distinct transcriptomic signatures in male and female *P. laevis* specimens from barbel associate with previous evidence of strong inverse sexual dimorphism in this species. In fact, mature females of *P. laevis* are about eight times as voluminous as males [64]. Without having carried out detailed measurements, we can confirm larger females than males for the animals analyzed here, especially in the animals from barbel. Such differences in size probably reflect that female morphology is tailored to high fecundity [46–48]. This was reflected in the present study in the enrichment of GO terms relating to gamete generation in genes the transcripts of which were particularly abundant in female vs. male *P. laevis* specimens from barbel (Fig. 6; Table 2). Annotations referring to developmental processes in the same group of genes might also refer to reproduction considering ongoing embryogenesis in hundreds or thousands of fertilized eggs floating in the female body cavity [5, 9, 49].

Among the genes with increased expression levels in male vs. female worms from barbel, linkage to reproduction was most evident in a strong enrichment of the GO cluster “spermatid development” (Fig. 7; Table 2). Yet, enrichment of GOs referencing cilia probably suggests the same since there is no body ciliation in acanthocephalans [65, 66]. Likewise, *P. laevis* lacks protonephridia which in some acanthocephalan taxa bear cilia [49, 67, 68]. Furthermore, potential derivatives of cilia with sensory function have been reported for few acanthocephalans [69, 70] but not for *P. laevis*. Not least, there were no functional categories highlighting enrichment of cilia-related genes in the transcriptomes of female worms, whether these were taken from barbel or eel. Thus, the male reproductive system, especially sperm production and storage, provides the most plausible explanation for increased frequencies of cilia-related GOs in male *P. laevis* individuals excised from barbel.

In accordance with previous evidence of glycogen metabolism and storage particles in acanthocephalans [31, 44, 45] strikingly many genes from glycolysis/gluconeogenesis and citrate cycle exhibited increased transcript numbers in male vs. female acanthocephalans from barbel (Table 1; Additional file 1: Fig. S1, S7). This might be due to enhanced intra-male competition for attractive attachment sites and fathering offspring [51, 53, 54]. In addition, male acanthocephalans are considered to play a more active role in mating than females [55–57]. Thus, the female appears to be primarily adapted to receiving the male and processing eggs, while the male is tailored to bringing spermatozoa to the females [71]. Consistently, the GO clusters “muscle contraction” and “myofibril assembly” were exclusively enriched in genes with higher transcript numbers in male vs. female worms from barbel. Increased male locomotion activity would additionally accord with the fact that the nervous system is more complex in male than female acanthocephalans [56, 72]. A correlate of this in the present study was the enrichment of a neurotransmitter-related GO cluster in genes with more transcripts in males vs. females from barbel (see also Additional file 1: Supplementary Note S1). Not least, enrichments of functional categories relating to photoreceptors and response to light (including *dac*, see below) in the transcriptomes of eye-less acanthocephalans (Fig. 7) might testify to eyed ancestors in the Gnathifera clade (for a discussion, see Additional file 1: Supplementary Note S1).

### Compromised energy metabolism and gametogenesis of *P. laevis* in a less suitable host, the eel

Reduced to collapsed signatures of sexual differentiation in the transcriptomes of *P. laevis* specimens from eel add to previous reports of arrested development and impaired reproduction in this host species [73, 74]. The specimens we collected from eel were flabby, whereas those from barbel were turgid. In addition, the testes were smaller in male worms collected from eel than those from barbel. Nevertheless, sexual maturation and reproduction sporadically occur in *P. laevis* specimens parasitizing the eel [59]. An approximation to maturity might be seen in one male and one female worm with transcriptomic profiles intermediate between their sex mates from both hosts (Figs. 1 and 2). In fact, the transcriptomic profiles of the male and female worms from eel were intermediary in PCA and distance matrix analyses, in stark contrast to the distinct patterns in their sex mates from barbel (Figs. 1 and 2).

It seems plausible to assume that the limitations in development in the eel reflect the inability of the worms to recruit sufficient energy. If so, the males seem to cope better than the females with the challenge as suggested by transcriptome-wide patterns (Fig. 3) and functional annotation analyses (Figs. 4, 6, 7 and 8; Additional file 1: Figs. S6–S9). Broad disturbance of acanthocephalan metabolism in the eel is probably coupled to a stress response as illustrated by enrichment of corresponding gene sets with functional terms referencing to “response to oxidative stress” in females (Fig. 8A), or “response to wounding” and “apoptosis” in males (Additional File 1: Fig. S9). Lastly, links to signaling pathways might be seen in the context of cell proliferation [75–79] and thus development or reproduction, but could also relate to immunological challenges *P. laevis* is exposed to (Additional file 1: Supplementary Note S1).

### The acanthocephalan model in relation to other pathogenic helminths

Differential transcriptional landscapes of sexes and developmental stages have been described in other parasitic helminths including roundworms (Nematoda) and tapeworms (Cestoda) amongst others [80–82]. However, the data seem to be particularly extensive for schistosomes (Trematoda, Digenea) [5]. In these endoparasites, interaction with the host immune response has left signatures in the transcriptomes [83], an aspect which we discuss in regard to *P. laevis* in Additional file 1: Supplementary Note S1. Furthermore, GO terms relating to energy metabolism were previously reported to be enriched in male schistosomes [83–86]. This aligns with present evidence of up-regulated energy metabolism especially in male acanthocephalans. Similarities were further traceable down to individual genes, as illustrated by the gene coding for dachshund protein (*dac*). In schistosomes, *dac* was contained in the female-biased GO term cluster “response to light” [83]. In the acanthocephalan *P. laevis*, *dac* showed elevated transcript levels in females vs. males from barbel – and in males vs. females from eel, underlining stronger disturbance of female gene regulation in the eel (not shown). However, there were also differences. For example, the GO term cluster “reproduction” was reported to be enriched in female schistosomes [83].

However, present GO analysis suggested high reproductive activity for male and female worms from barbel. The picture changed in worms from eel, where GO term enrichment indicated slight up-regulation of genes involved in reproduction in male *P. laevis* specimens, underlining their capability to cope better with the environment provided by the eel. In adults of *S. mansoni*, “neurological process” was another functional category found to be overrepresented in females [83]. Contrarywise, connections to the nervous system were more prominent in male than female worms from barbel, thus corroborating a more active role of male acanthocephalans in reproduction [55–57]. These differences could reflect specificities of the study designs: In previous investigations on schistosomes [83–85], the comparison was made between the sexes or between developmental stages on the track of the lifecycle (eggs, schistosomules, adults). In contrast, we have compared *P. laevis* transcriptomes between developmentally delayed adults from a less suitable host offside the lifecycle and developmentally unhalted adults on the track of the lifecycle. Notwithstanding the usefulness of the approaches pursued before, we believe to have demonstrated that a “within/offside the lifecycle perspective” can elucidate the molecular causes behind the developmental arrest of parasites. In addition, we think this approach has enabled interesting insights regarding worms that stand between the ‘normal’ phenotypes in suitable and unsuitable hosts. More importantly, the approach, applied here to acanthocephalans, might bring us closer to define novel starting points for the development of an effective and sustainable parasite control. The need for this is high in acanthocephalans, as they are increasingly appearing as a pest in fish aquaculture [20, 22–26, 87], but also in other endoparasitic helminths. In fact, the dispersal of established anthelmintic agents into the environment is problematic due to their limited specificity. The dimension of this problem might be highlighted by the use of current anthelmintics against phylogenetically distant metazoans [15] and their potential as cytostatics in cancer therapy [88].

## Conclusions

It has previously been shown that some acanthocephalans do not reach full reproductive state in accidental and paratenic hosts [50, 89, 90], but the molecular background of this phenomenon was little known. Here we present first-time evidence on the functional level for halted sexual maturation of a parasitic species when established in a non-definitive host. By comparison of transcript abundances between worms from a definitive (common barbel) and a less suitable host (European eel), we unraveled that disturbance of energy metabolism appears to prevent the parasites from reaching full maturity in the eel. Accordingly, new active substances to be developed should target the parasite’s energy metabolism [44]. Here, it may prove worthwhile to consider the proteins listed in Table 1. Beyond acanthocephalans, the present results demonstrate that comparative transcriptome analysis of mature parasites from a definitive host and developmentally arrested parasites from a, in terms of maturation, less suitable host provides a useful avenue for elucidating the molecular background of parasite-host specificity. In addition, the “within/offside the lifecycle perspective” taken here might prove useful for developing novel strategies in the control of acanthocephalans and other parasitic helminths.

## Methods

### Samples and sequencing

Fishes were caught in a gravel pit near Gimbsheim, Germany (barbel) and in the river Weser near Gieselwerder, Germany (eel) in 2006–2015 (Additional file 1: Table S1). Acanthocephalans were excised from guts immediately after fishes were sacrificed. After excision, the worms were transferred into cooled physiological saline, in which they freed themselves from host tissue and mucus by their movements. Since all this was done at summer temperatures, rapid processing was necessary for maintaining high RNA quality. Therefore, we have concentrated on a synoptic recording of the finding circumstances and morphological parameters. Upon transportation in cooled physiological saline to the laboratory, any residual debris was removed from the animals with forceps before they were frozen at -80 °C in dry state. Upon gentle thawing the worms were sexed based on the presence/absence of tandem-arranged testes, evertible bursa copulathrix, cement glands, female reproductive tract, copulatory caps etc. Following this, the worms were overlaid with TriReagent (Invitrogene) and minced with micro-pestles. Extraction of RNA was done in accordance to the manufacturer’s protocol. Pelleted RNA was eluted in HPLC grade H_2_O. Subsequently, we determined the concentration of RNA solutions by Qubit assay and validated RNA quality by gel electrophoresis. Library construction and sequencing on an Illumina HiSeq 2500 (75 bp, single-end reads, 30 million reads per sample) was performed by a commercial provider. We analyzed altogether 20 thorny-headed worms (*P. laevis*), with 10 specimens (five males and five females) from common barbel and 10 specimens (five males and five females) from European eel. In light of recent evidence that *P. laevis* may be a collective species [91], we follow others and here consider the individuals analyzed to represent *P. laevis sensu lato* [92]. The same applies to the individuals from which we had previously generated a transcriptome assembly that was used as a reference in the present study [58].

### Data processing

We trimmed adapter sequences and low quality parts of the reads (ILLUMINACLIP:2:30:10, LEADING:3, TRAILING:3, SLIDINGWINDOW:4:15, MINLEN:40) with Trimmomatic v0.39 [93]. All datasets were quality-checked with FastQC v0.11.9 [94] before and after quality processing.

As reference we used the *P. laevis* transcriptome published recently under NCBI GenBank accession number GIBA00000000.1 [58]. This transcriptome shotgun assembly was generated by Trinity v2.4.0 [95] from male, female and juvenile *P. laevis* specimens. To check for congruence of our datasets with the reference transcriptome we mapped all datasets with BBMap v38.73 [96]. For all datasets 92–96% of reads mapped to the reference transcriptome, thus illustrating the suitability of the reference for downstream analyses.

### Comparative analysis of transcript abundances

Transcript quantification was done with the RSEM v1.3.3 software package [97] and the reference transcriptome described above. We applied Bowtie 2 v2.4.1 [98] mapping with settings optimized and implemented for RSEM downstream analysis. The “rsem-calculate-expression” script was applied with -calc-ci option for the inference of confidence intervals during calculation of relative transcript abundances.

Differential gene expression analyses were carried out with the Bioconductor package DESeq2 v1.28.1 [99] in R [100, 101]. Since we are interested in gene expression values rather than transcript expression values and furthermore DESeq2 requires integers as input, we summed up read counts from transcript variants for single genes (based on Trinity annotations). Integers were then used for differential expression analysis with DESeq2, applying the log fold-change shrinkage method “ashr” [102]. All analyses are based on the adjusted p-values (*padj*), generated by false discovery rate correction by the Benjamini and Hochberg method on the Wald test attained p-values. MA-plots and principal component analysis were carried out in DESeq2.

### Functional annotation of genes with differential transcript abundances

For retrieving of gene identifiers, we searched for matches of *P. laevis* genes in next-related model systems, i.e., the nematode *Caenorhabditis elegans* and the hexapod *Drosophila melanogaster*. As more genes could be matched in the fruit fly (N = 5,098) than in the equally distant nematode (N = 3,146), we focused on FlyBase gene identifiers (FBgn’s). These had been retrieved by BLASTX [103] searches with e-value ≤ 1e-05 against the full set of *D. melanogaster* genes (‘dmel-all-gene-r6.36’, retrieved from flybase.org [104]). Enrichment analyses of functional annotation terms were carried out with the online toolbox Metascape v3.5 [105] applying default settings. The full set of annotated transcripts was used as the backbone to test against. The KEGG pathway 00010 “glycolysis/gluconeogenesis” was analyzed in depth by manually mapping all differentially expressed genes onto the pathway downloaded from the GenomeNet database (genome.jp; accessed 2021-10-01).

Results by Metascape were verified in an analog approach using the plugin BiNGO v3.0.3 [106] in the network visualization platform Cytoscape v3.8.2 [107]. BiNGO analysis included a hypergeometric test with Benjamini & Hochberg false discovery rate detection at a significance level of 0.05 and the same backbone as for Metascape.

## Supplementary Information

**Additional file 1.** Supplementary Note S1. Table S1. Figures S1–S9.

**Additional file 2.** Table S2.

## Data Availability

The datasets analyzed in the current study are available in the EMBL Nucleotide Sequence Database (ENA) repository under the accession number PRJEB47442 (ERS7302868-87, specified in Additional file 1: Table S1).
