# Supplementary material for: Host-dependent impairment of parasite development and reproduction in the acanthocephalan model
Source: Cell Biosci. 2022 May 31;12:75. doi: 10.1186/s13578-022-00818-2 (PMC9153150; doi:10.1186/s13578-022-00818-2)
Supplement: Supplementary file 1 — Additional file 1. Supplementary Note S1. Table S1. Figures S1–S9. [file 13578_2022_818_MOESM1_ESM.pdf]

Supplementary Material to “Host-dependent impairment of parasite development and reproduction in the acanthocephalan model”

Hanno Schmidt, Katharina Mauer, Thomas Hankeln, Holger Herlyn

Supplementary Notes

Supplementary Note S1 2

Supplementary Tables

Table S1 – Datasets 4
Table S2 – Differentially expressed genes 5

Supplementary Figures

Figure S1 – *P. laevis* genes involved in glycolysis/gluconeogenesis showing increased transcript abundances in males vs. females from barbel 6
Figure S2 – GO terms enriched in genes with elevated transcript levels in female vs. male worms from barbel 7
Figure S3 – GO terms enriched in genes with elevated transcript levels in male vs. female worms from barbel 8
Figure S4 – GO terms enriched in genes with elevated transcript levels in female vs. male worms from eel 9
Figure S5 – GO terms enriched in genes with elevated transcript levels in male vs. female worms from eel 10
Figure S6 – Genes with reduced transcript abundances in female acanthocephalans from eel vs. barbel 11
Figure S7 – Genes with elevated transcript abundances in female acanthocephalans from eel vs. barbel 12
Figure S8 – Genes with reduced transcript abundances in male acanthocephalans from eel vs. barbel 13
Figure S9 – Genes with elevated transcript abundances in male acanthocephalans from eel vs. barbel 14

References 15

Supplementary Notes

Supplementary Note S1

Enrichment of genes related to eye development and potential links to eye-less acanthocephalans

Gene Ontology (GO) analyses suggested connections to “compound eye development” in female vs. male and “phototransduction” in male vs. female acanthocephalans from barbel, respectively. This is partly due to the fact that we had used gene IDs of homologs in eyed *D. melanogaster* for GO analyses of *P. laevis* transcripts. Nevertheless, eyes probably existed prior to the divergence of Bilateria [1] and some of the genes originally involved in eye development and functioning may well have persisted in the genome of *P. laevis*. Indeed, we were able to trace potentially homologous sequences coding for two master transcription factors for eye development [*Pax6*/*eyeless* (acc. no. AAX52512.1) and *Six3*/*optix* (acc. no. NP_001260793.1)] in the reference transcriptome of *P. laevis* (tblastn hits with e-value ≤ 1e-50). In line with this, evidence for rhodopsin-associated enzymes and transmembrane receptors of the rhodopsin family have previously been reported for the *P. laevis* transcriptome [2]. Likewise, transmembrane receptors of the rhodopsin family have been predicted for a bdelloid (SwissProt: B2L3H7_PHIRO), and the "eyespot" in monogononts is assumed to contain rhodopsin [3]. Moreover, a pair of eyespots is present in *Limnognathia maerski* (Micrognathozoa) [4], the probable sister-taxon of the clade including wheel animals and acanthocephalans (reviewed in [5]). In additon, arrow-worms (Chaetognatha), which may also belong to the closer phylogenetic relationship, possess a pair of compound eyes the ocelli of which presumably contain rhodopsin-like photopigments [6, 7]. Accordingly, rhodopsin-mediated phototransduction could have existed in the last common ancestor of Gnathifera from which it potentially was passed on to its descendants. Still, the conservation of such genes may be more indicative of nutrition in eye-less acanthocephalans as discussed elsewhere [2].

Host-dependent immunological challenges and hints for host-parasite crosstalk

As outlined above, the eel does not provide as good living conditions for *P. laevis* as does the barbel. Obviously, this is not because the European eel has nothing to offer to endoparasites as illustrated by the acanthocephalan *Acanthocephalus anguillae* and the swim-bladder worm *Anguillicoloides crassus* (Nematoda, Dracunculoidea) that both exploit the European eel [8-10]. Rather, the deeper reason for arrested development of *P. laevis* in the eel could be a stronger host-parasite interaction [11]. In the present study, footprints of increased *Wnt* signaling in male vs. female, and *notch* signaling in female vs. male worms from eel could point to the particular immunological challenge *P. laevis* is facing in this host. In support of this view, *Wnt* signaling has been implicated in T cell inflammation and orchestration of immune response to parasites [12, 13]. Likewise, *notch* signaling regulates T

lymphocyte processes in host defense [14] and clearance of gastrointestinal helminth parasites [15] in other systems. Compared to this, the challenges worms have to cope with in barbel seem to be rather unspecific. In fact, the GO cluster "innate immune system" enriched in genes with higher transcript abundances in males vs. females and "signaling by Rho GTPases" in females vs. males could be indicative of a broad spectrum of immunological responses [16]. These clues to the host immune response unlikely reflect contamination of the *P. laevis* samples since we had extracted the RNAs sequenced from decapitated worms to which no host tissue was attached. In addition, mapping rates to a reference transcriptome of *P. laevis* were high (92-95%) for all 20 datasets analyzed here. Furthermore, the reference transcriptome had been filtered for potential contamination of the sample with cyprinid tissue [2], and mismapping of host reads to parasite sequences seems very unlikely given the high age of their split of > 600 million years [17]. However, the enrichment of the signaling pathways mentioned can also be understood as an indication of cell proliferation and developmental processes [18-22].

Supplementary Tables

Table S1 – Datasets

§ = *Barbus barbus* caught in June 2006 in a gravel pit near Gimbsheim, Germany, # = *Anguilla anguilla* caught in June 2014 and 2015 in River Weser near Gieselwerder, Germany.

Sample	Group	Raw reads	% Clean reads	% Mapped reads	ENA accession number
R3	female worms from barbel§	32,425,723	99.6	95.7	ERS7302868
R4	female worms from barbel§	33,231,064	99.6	95.6	ERS7302869
R5	female worms from barbel§	31,892,176	99.6	96.3	ERS7302870
R6	female worms from barbel§	29,173,941	98.9	95.5	ERS7302871
R7	female worms from barbel§	33,092,559	99.1	96.7	ERS7302872
R9	male worms from barbel§	31,062,589	99.6	96.4	ERS7302873
R10	male worms from barbel§	34,254,801	99.6	96.2	ERS7302874
R11	male worms from barbel§	29,621,019	98.9	95.8	ERS7302875
R13	male worms from barbel§	32,964,249	99.6	96.4	ERS7302876
R14	male worms from barbel§	34,931,058	99.3	96.8	ERS7302877

R16	female worms from eel#	35,222,695	98.9	97.0	ERS7302878
R17	female worms from eel#	30,331,587	98.9	97.1	ERS7302879
R18	female worms from eel#	23,314,674	99.1	95.3	ERS7302880
R19	female worms from eel#	31,021,260	98.9	95.3	ERS7302881
R20	female worms from eel#	33,606,218	98.8	96.9	ERS7302882
R24	male worms from eel#	39,213,452	98.8	95.3	ERS7302883
R25	male worms from eel#	34,622,964	98.9	94.5	ERS7302884
R26	male worms from eel#	31,167,324	98.9	95.0	ERS7302885
R27	male worms from eel#	35,015,790	98.9	95.4	ERS7302886
R28	male worms from eel#	35,176,073	99.6	95.4	ERS7302887
**mean**		**32,567,061**	**99.2**	**95.9**	

Table S2 – Differentially expressed genes

The table reports for all pairs of comparison genes showing differential transcript abundances. Only genes that could be annotated by homology search via BLASTX are included. Table S2 is available in Additional File 2 in Excel spreadsheet format.

Supplementary Figures

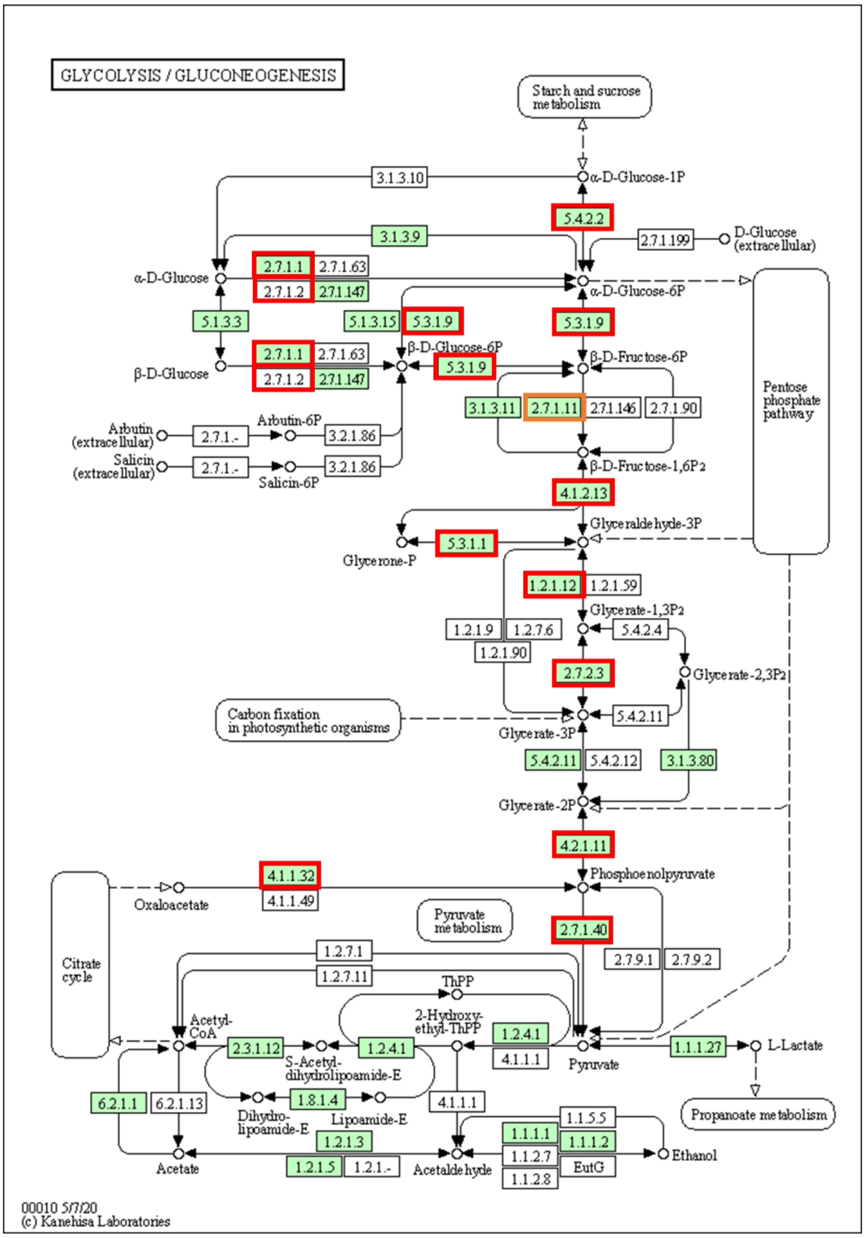

Figure S1 – *P. laevis* genes involved in glycolysis/gluconeogenesis showing increased transcript abundances in males vs. females from barbel

Eleven genes from KEGG pathway 00010 were found to have significantly more transcripts in male vs. female *P. laevis* from barbel. Green filling marks enzymes in *Drosophila melanogaster* as the phylogenetically relatively close reference species taken here; unfilled boxes represent enzymes that are specific to other taxa. Red framing indicates genes exhibiting significantly higher transcript numbers in male vs. female worms from barbel, orange framing indicates significantly higher abundances in female vs. male worms from the same host. Notably, the two enzymes linking glycolysis/gluconeogenesis with citrate cycle (EC:4.1.1.32 and EC:6.4.1.1, not included in the presented pathway depiction) were up-regulated in males vs. females as well.

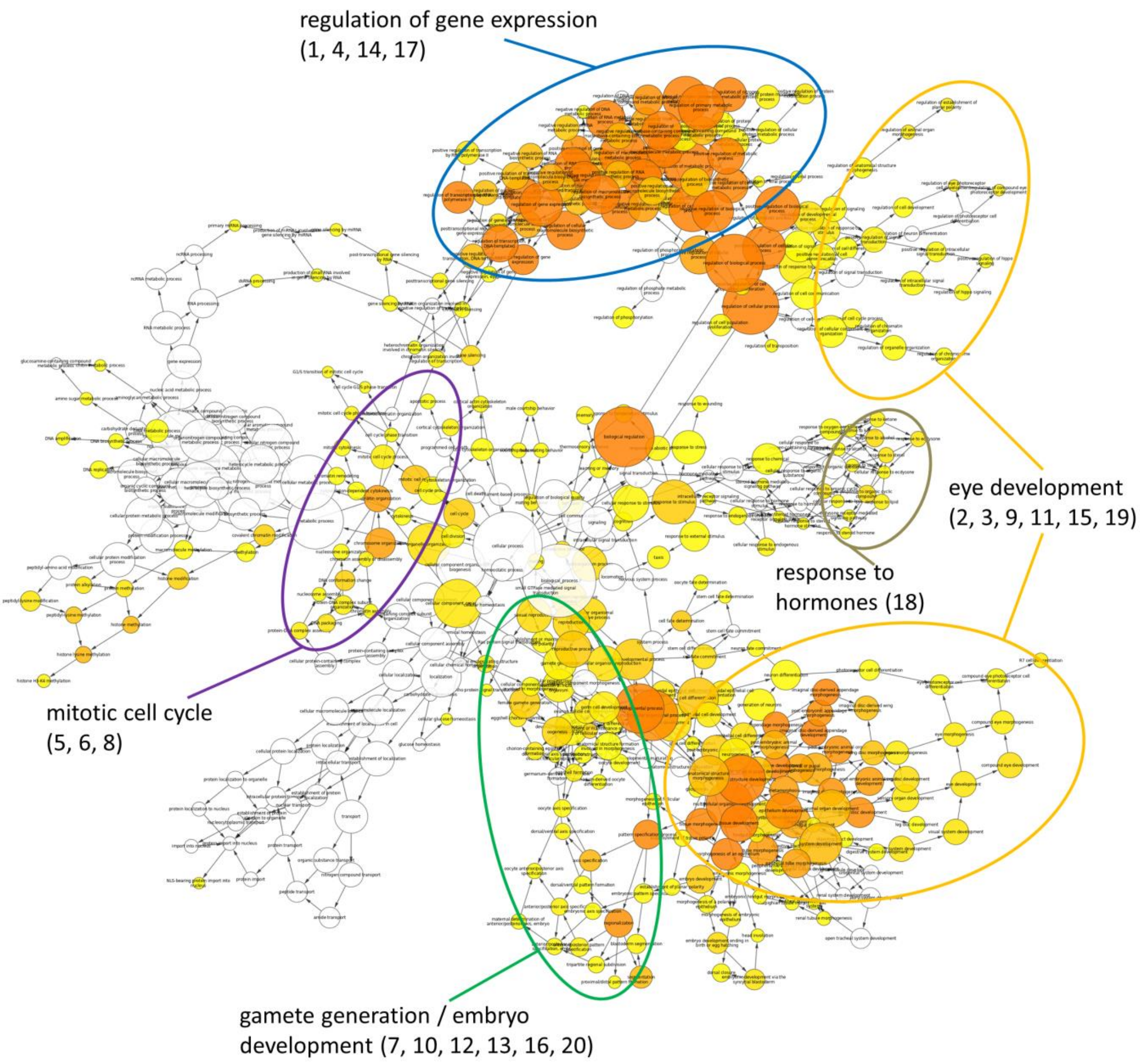

Figure S2 – GO terms enriched in genes with elevated transcript levels in female vs. male worms from barbel

Displayed are results from Gene Ontology (GO) enrichment analysis in BiNGO (Cytoscape). Colors refer to statistical significance of enrichment; the darker the orange, the lower the FDR-adjusted p-value. Ovals sum GO terms by higher biological processes. Numbers behind category names refer to the numbers in Fig. 5A (GO enrichment analysis with Metascape). All of the 20 groups were found.

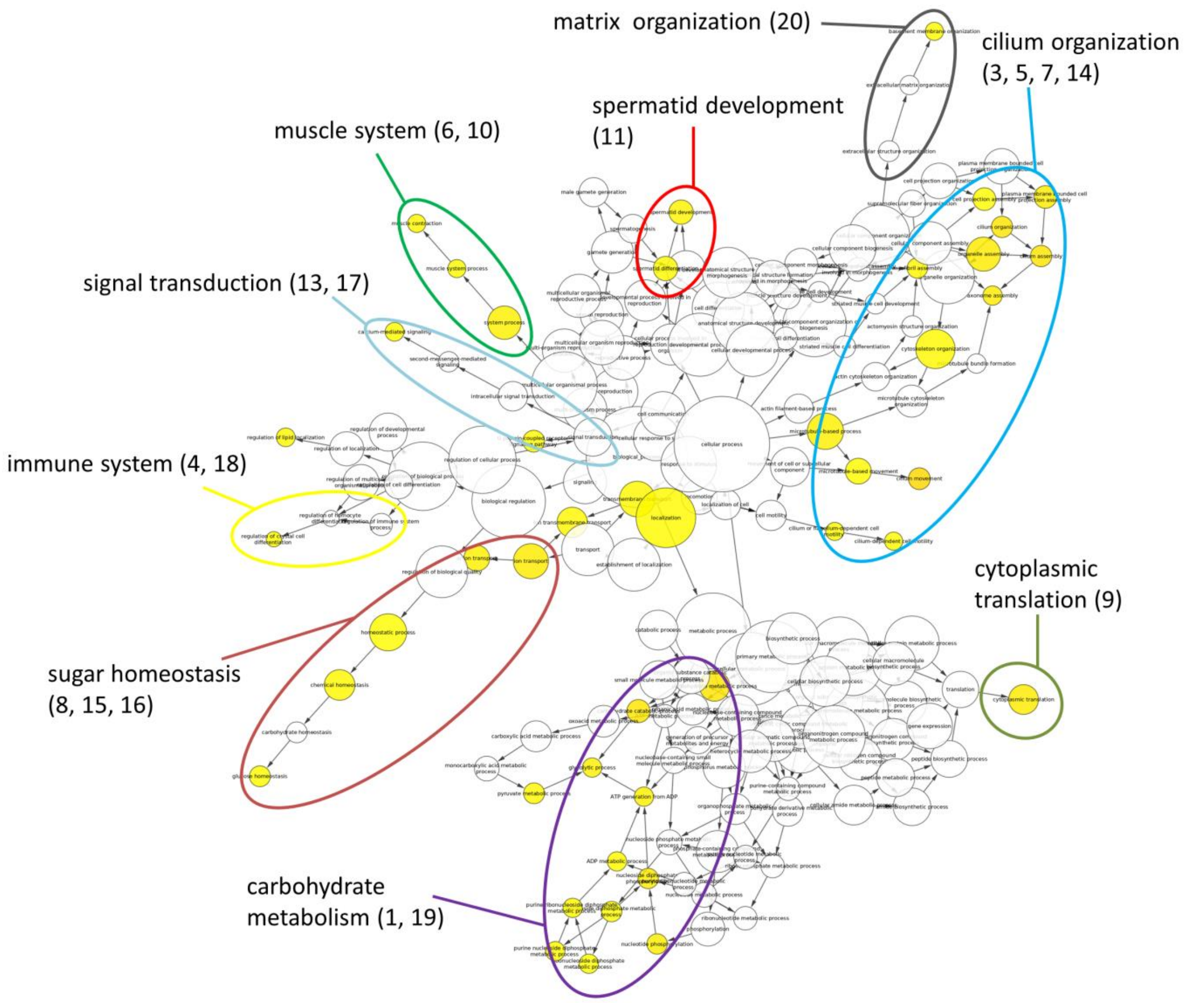

Figure S3 – GO terms enriched in genes with elevated transcript levels in male vs. female worms from barbel

Displayed are results from Gene Ontology (GO) enrichment analysis in BiNGO (Cytoscape). Colors refer to statistical significance of enrichment; the darker the orange, the lower the FDR-adjusted p-value. Ovals sum GO terms by higher biological processes. Numbers behind category names refer to the numbers in Fig. 5B (GO enrichment analysis with Metascape). 18 out of 20 groups were found.

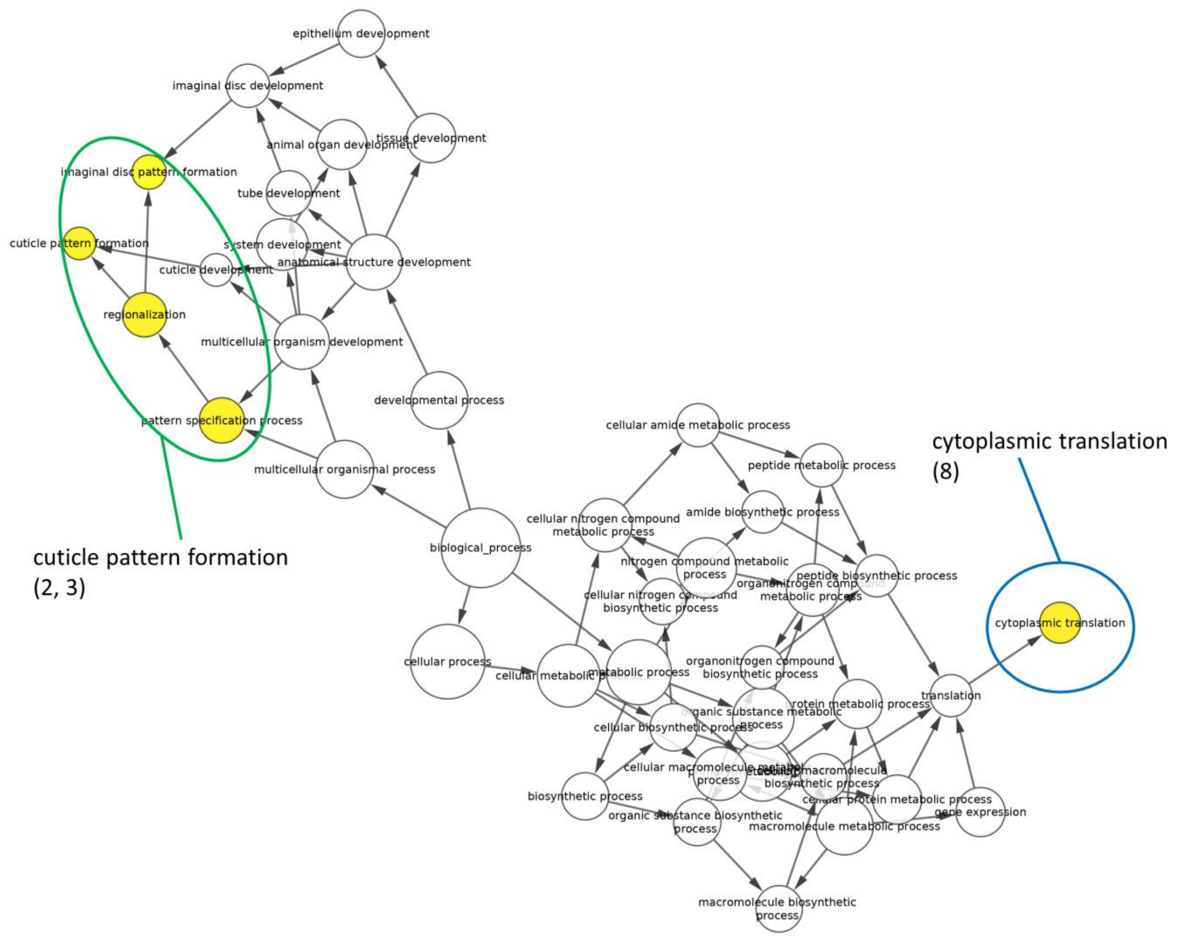

Figure S4 – GO terms enriched in genes with elevated transcript levels in female vs. male worms from eel

Displayed are results from Gene Ontology (GO) enrichment analysis in BiNGO (Cytoscape). Colors refer to statistical significance of enrichment; the darker the orange, the lower the FDR-adjusted p-value. Ovals sum GO terms by higher biological processes. Numbers behind category names refer to the numbers in Fig. 6A (GO enrichment analysis with Metascape). 3 out of 9 groups were found.

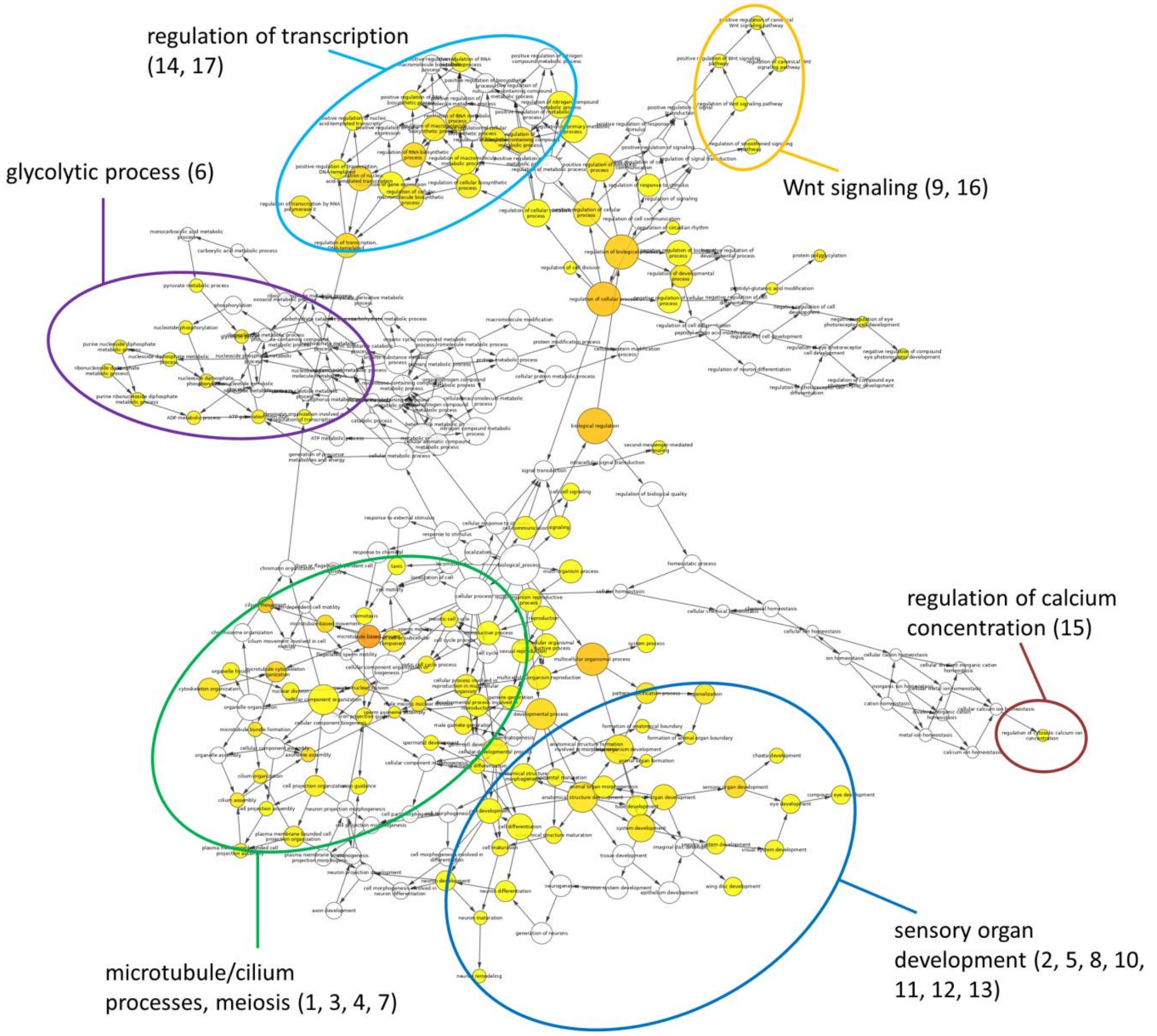

Figure S5 – GO terms enriched in genes with elevated transcript levels in male vs. female worms from eel

Displayed are results from Gene Ontology (GO) enrichment analysis in BiNGO (Cytoscape). Colors refer to statistical significance of enrichment; the darker the orange, the lower the FDR-adjusted p-value. Ovals sum GO terms by higher biological processes. Numbers behind category names refer to the numbers in Fig. 6B (GO enrichment analysis with Metascape). 17 out of 20 groups were found.

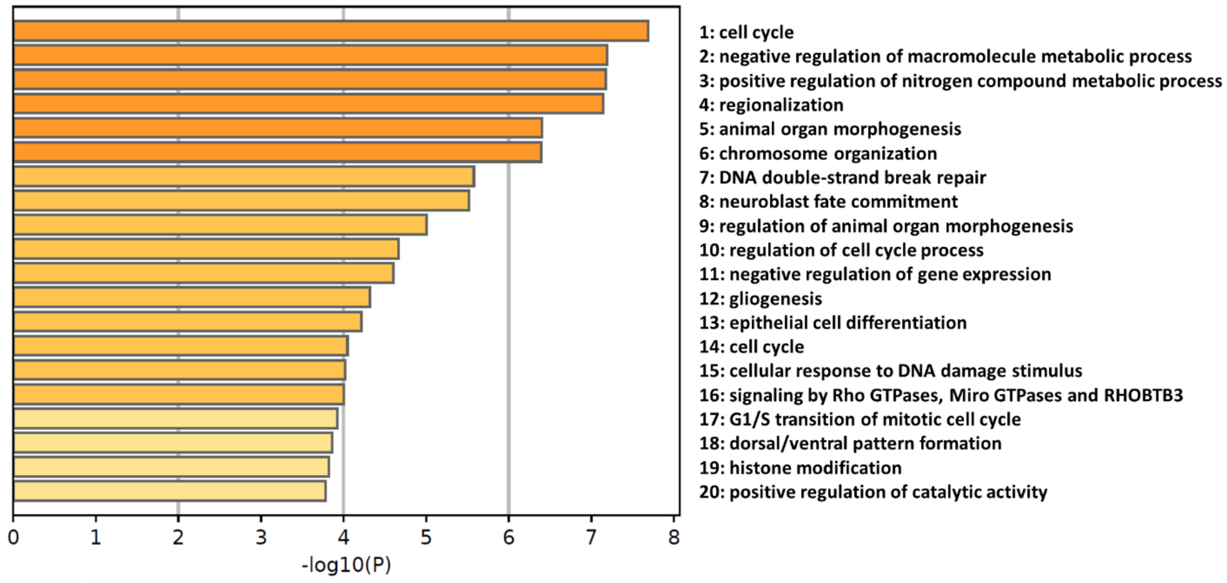

Figure S6 – Genes with reduced transcript abundances in female acanthocephalans from eel vs. barbel

Shown are the functional categories with highest significance for enrichment.

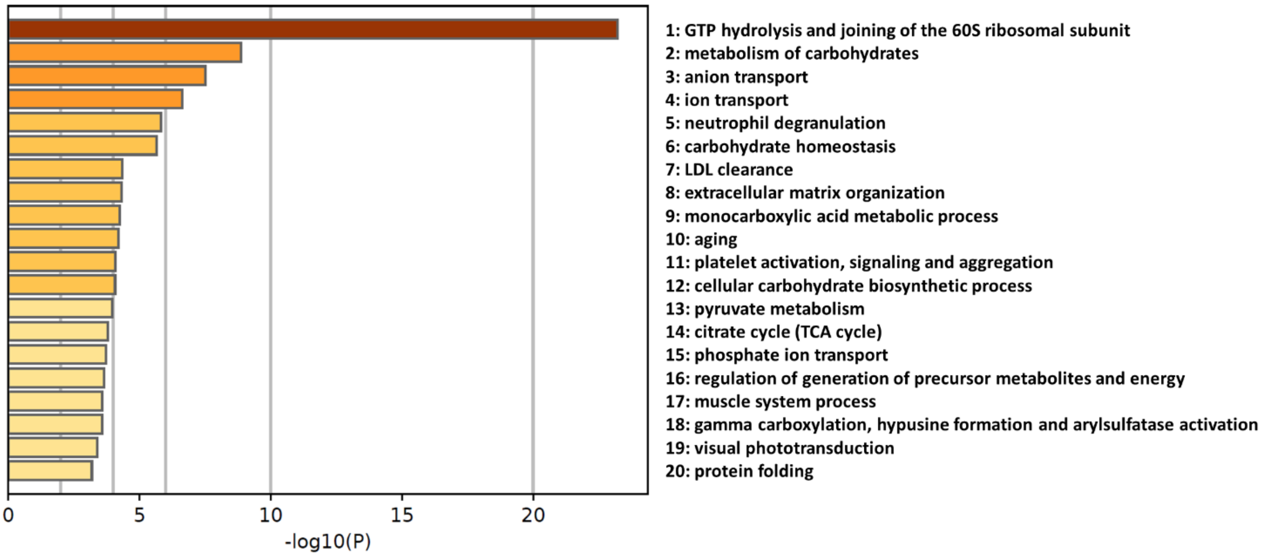

Figure S7 – Genes with elevated transcript abundances in female acanthocephalans from eel vs. barbel

Shown are the functional categories with highest significance for enrichment.

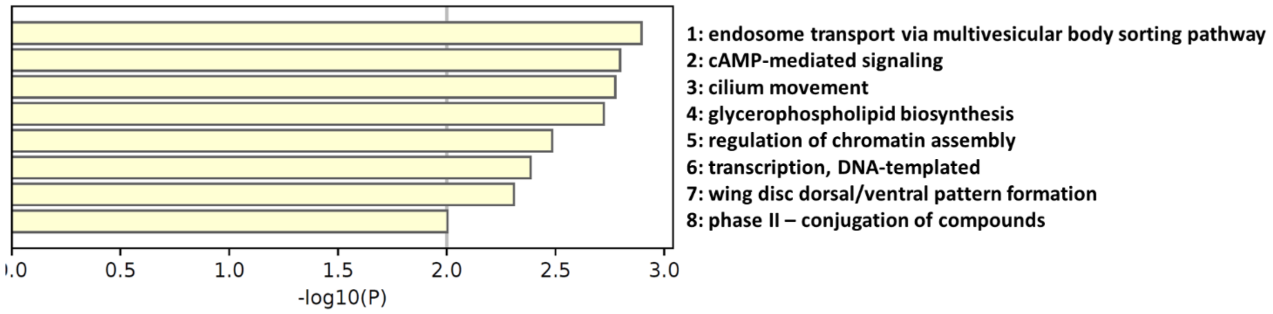

Figure S8 – Genes with reduced transcript abundances in male acanthocephalans from eel vs. barbel

Shown are the functional categories with highest significance for enrichment.

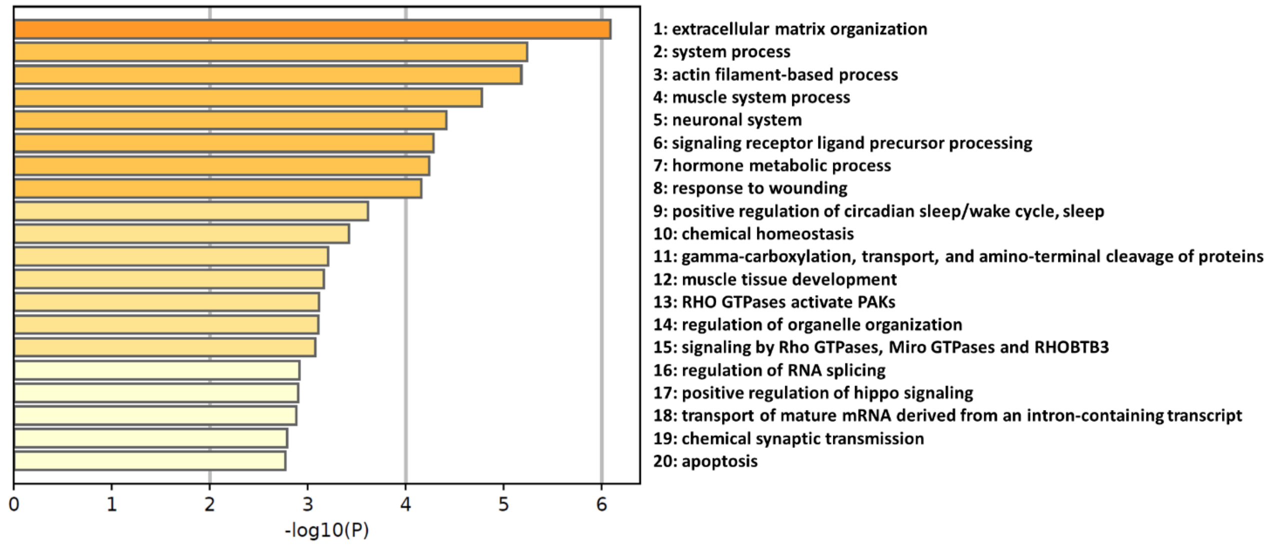

Figure S9 – Genes with elevated transcript abundances in male acanthocephalans from eel vs. barbel

Shown are the functional categories with highest significance for enrichment.

References

1. Gehring WJ. New perspectives on eye development and the evolution of eyes and photoreceptors. J Hered. 2005;96:171-84.
2. Mauer K, Hellmann SL, Groth M, Frobius AC, Zischler H, Hankeln T, Herlyn H. The genome, transcriptome, and proteome of the fish parasite *Pomphorhynchus laevis* (Acanthocephala). PLoS One. 2020;15(6):e0232973.
3. Kim H-J, Sawada C, Rhee J-S, Lee J-S, Suga K, Hagiwara A. Nutritional effects on the visual system of the rotifer *Brachionus plicatilis sensu stricto* (Rotifera: Monogononta). J Exp Mar Biol Ecol. 2014;460:177-83.
4. Funch P, Sørensen MV, Obst M. On the phylogenetic position of Rotifera – have we come any further? Hydrobiologia. 2005;546:11-28.
5. Herlyn H. Thorny-headed worms (Acanthocephala): jaw-less members of jaw-bearing worms that parasitize jawed arthropods and jawed vertebrates. In: De Baets K, Huntley JW, editors. The Evolution and Fossil Record of Parasitism - Identification and Macroevolution of Parasites. Cham, Switzerland: Springer; 2021. p. 273-313.
6. Marlétaz F, Peijnenburg KTCA, Goto T, Satoh N, Rokhsar DS. A new spiralian phylogeny places the enigmatic arrow worms among gnathiferans. Curr Biol. 2019;29(2):312-18.
7. Perez Y, Müller CHG, Harzsch S. The Chaetognatha: An anarchistic taxon between Protostomia and Deuterostomia. In: Wägele JW, Bartolomaeus T, editors. Deep Metazoan Phylogeny: The Backbone of the Tree of Life. De Gruyter; 2014. p. 49-77.
8. Taraschewski H, Moravec F, Lamah T, Anders K. Distribution and morphology of two helminths recently introduced into European eel populations: *Anguillicola crassus* (Nematoda, Dracunculoidea) and *Paratenuisentis ambiguus* (Acanthocephala, Tenuisentidae). Dis Aquat Org. 1987;3:167-76.
9. Kennedy CR, Berrilli F, Di Cave D, De Liberato C, Orecchia P. Composition and diversity of helminth communities in eels *Anguilla anguilla* in the River Tiber: long-term changes and comparison with insular Europe. J Helminthol. 1998;72:301-06.
10. Emde S, Rueckert S, Kochmann J, Knopf K, Sures B, Klimpel S. Nematode eel parasite found inside acanthocephalan cysts - a "Trojan horse" strategy? Parasites Vectors. 2014;7:504.
11. Behnke JM, Parish HA. *Nematospiroides dubius*: arrested development of larvae in immune mice. Exp Parasitol. 1979;47:116-27.
12. Li X, Xiang Y, Li F, Yin C, Li B, Ke X. Wnt/β-catenin signaling pathway regulating T cell-inflammation in the tumor microenvironment. Front Immunol. 2019;10:2293.

13. Volpini X, Ambrosio LF, Brajín A, Brugo MB, Aoki MP, Rivarola HW, Alfonso F, Fozzatti L, Cervi L, Motran CC. Wnt signaling plays a key role in the regulation of the immune response and cardiac damage during *Trypanosoma cruzi* infection. ACS Infect Dis. 2021;7(3):566-78.

14. Brandstadter JD, Maillard I. Notch signalling in T cell homeostasis and differentiation. Open Biol. 2019;9(11):190187.

15. Webb LM, Oyesola OO, Früh SP, Kamynina E, Still KM, Patel RK, Peng SA, Cubitt RL, Grimson A, Grenier JK *et al*. The Notch signaling pathway promotes basophil responses during helminth-induced type 2 inflammation. J Exp Med. 2019;216(6):1268-79.

16. Masri RE, Delon J. RHO GTPases: from new partners to complex immune syndromes. Nat Rev Immunol. 2021;21:499-513.

17. dos Reis M, Thawornwattana Y, Angelis K, Telford MJ, Donoghue PCJ, Yang Z. Uncertainty in the timing of origin of animals and the limits of precision in molecular timescales. Curr Biol. 2015;25:2939-50.

18. Provenzano PP, Keely PJ. Mechanical signaling through the cytoskeleton regulates cell proliferation by coordinated focal adhesion and Rho GTPase signaling. J Cell Sci. 2011;124(8):1195-205.

19. Fre S, Pallavi SK, Huyghe M, Louvard D. Notch and Wnt signals cooperatively control cell proliferation and tumorigenesis in the intestine. Proc Natl Acad Sci USA. 2009;106(15):6309-14.

20. Gönczy P. Mechanisms of asymmetric cell division: flies and worms pave the way. Nat Rev Mol Cell Biol. 2008;9:355-66.

21. Greenwald I. LIN-12/Notch signaling: lessons from worms and flies. Genes Dev. 1998;12:1751-62.

22. Schlessinger K, Hall A, Tolwinski N. Wnt signaling pathways meet Rho GTPases. Genes Dev. 2009;23:265-77.
